# Brassinosteroid controls leaf air space patterning non-cell autonomously by promoting epidermal growth

**DOI:** 10.1242/dev.205110

**Published:** 2026-05-26

**Authors:** James M. Fitzsimons, Ana B. Rock, Richard L. De Falbe, Samantha Fox, Chris D. Whitewoods

**Affiliations:** ^1^ Sainsbury Laboratory, University of Cambridge, 47 Bateman Street, Cambridge CB2 1LR, UK; ^2^ Department of Cell and Developmental Biology, John Innes Centre, Norwich Research Park, Norwich NR4 7UH, UK

**Keywords:** Plant development, Air space, Mesophyll, Leaf morphogenesis, Brassinosteroid, Non-cell autonomous

## Abstract

Plant leaves contain a complex network of intercellular air spaces, which allow efficient photosynthesis. However, how leaf air spaces are patterned is poorly understood. It has been proposed for almost a century that air spaces form by faster-growing epidermal tissue pulling slower growing mesophyll cells apart, but this has never been tested. Here, we characterise air space morphogenesis throughout in the first leaf of *Arabidopsis thaliana* and show that the plant hormone brassinosteroid is required for air space expansion in the palisade, but not in the spongy mesophyll. We also show that epidermal brassinosteroid perception is sufficient to promote air space expansion in the palisade non-cell autonomously and propose that this non-cell-autonomous effect is due to altered epidermal growth. To test whether epidermal growth affects air space patterning we reduce growth specifically in the epidermis using inducible expression of the growth repressor BIG BROTHER and show that an epidermal growth restriction reduces air space expansion in the palisade mesophyll. Overall, we propose that brassinosteroid signalling promotes growth in the epidermis to pattern air spaces in the palisade mesophyll.

## Teaser

**Summary:** Up to 70% of leaf volume is intercellular air space. This paper shows that the plant hormone brassinosteroid acts in the epidermis to promote air space morphogenesis non-cell autonomously.

## INTRODUCTION

The size and complexity of organisms are limited by their ability to obtain nutrients from their environment by diffusion (Haldane, 1927). To overcome this limitation, multicellular animals and plants have evolved internal structures with large surface areas to increase nutrient absorption. The convoluted air spaces found within the lungs of animals and the leaves of plants are key examples of this. Air spaces bring internal cells into contact with the atmosphere and mediate the efficient exchange of carbon dioxide and oxygen gases necessary for respiration and photosynthesis. In animals, the developmental and molecular mechanisms of air space formation within lungs are well studied, with several genes interacting to control elongation and branching of an air-filled tissue tube (Metzger et al., 2008; Nikolić et al., 2018). However, in plants air space development is poorly understood. This is all the more striking as air spaces form up to 70% of leaf volume (Earles et al., 2018) and affect leaf photosynthetic efficiency (Lehmeier et al., 2017; Mathers et al., 2018).

**Fig. 1.**
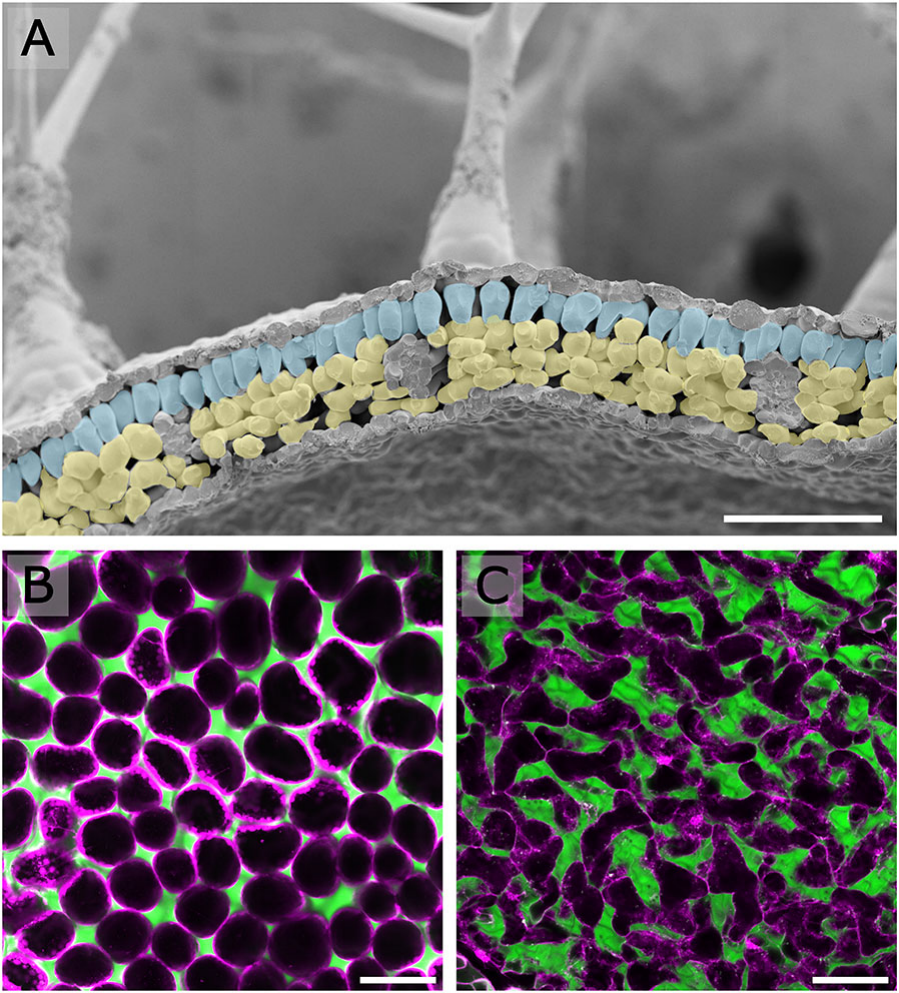
***A. thaliana* leaves contain palisade and spongy mesophyll cells surrounded by intercellular air space.** (A) Transverse section of a mature *A. thaliana* leaf showing palisade mesophyll (false coloured blue) and spongy mesophyll (false coloured yellow) visualised by freeze-fracture Cryo-SEM. (B,C) Palisade (B) and spongy (C) mesophyll tissue visualised with Nile red dye. Air spaces are green, cell outlines magenta. Scale bars: 100 µm.

Leaf air spaces form by cell separation at multicellular junctions (Jeffree et al., 1986; Pyke et al., 1991; Kalve et al., 2014) rather than by cell death as in roots of many species (Evans, 2004). These smaller air spaces expand and join to form an interconnected network throughout the leaf, patterned precisely in three dimensions. The upper half of many leaves contains densely packed palisade mesophyll cells with only small air spaces (Fig. 1A,B) whereas the lower half contains lobed spongy mesophyll cells with larger air spaces (Fig. 1A,C). The largest spaces are positioned adjacent to stomata. This arrangement is thought to maximise light harvesting in the top half (Terashima and Inoue, 1984) and gas exchange and light scattering in the lower half to increase overall photosynthetic efficiency (DeLucia et al., 1996; Smith et al., 1997).

Air space formation and patterning has been proposed to be controlled either cell autonomously in mesophyll cells [by local loss of cell adhesion (Jeffree et al., 1986) and increased growth of cell walls adjacent to air spaces (expansigeny) (Zhang et al., 2021; Treado et al., 2022)], or non-cell autonomously by a mechanical mechanism where the epidermis grows faster than the underlying mesophyll and pulls mesophyll cells apart to form and expand air spaces (Avery, 1933). There is some support for both hypotheses: recent live imaging and computational modelling has shown that air spaces in the spongy mesophyll expand by expansigeny, supporting the cell autonomous hypothesis in spongy mesophyll cells (Zhang et al., 2021; Treado et al., 2022). Reduction of epidermal growth by epidermal expression of the cell-cycle inhibitor *KIP-RELATED PROTEIN 1* (*KRP1*) also decreases mesophyll porosity in the palisade, suggesting that epidermal growth may control air space patterning non-cell autonomously (Lehmeier et al., 2017). Periclinal chimeras between *Nicotiana* species with different leaf sizes also support a role for the epidermis controlling mesophyll patterning (Marcotrigiano, 2010): although porosity was not measured, in these data mesophyll cell number scales with epidermal size, rather than remaining fixed based on mesophyll genotype. This suggests that an intercellular signal (molecular or mechanical) co-ordinates mesophyll and epidermal patterning. However, no molecular regulators of leaf air space patterning have yet been identified.

We have recently shown that the plant hormone brassinosteroid (BR) promotes epidermal growth in plant stems, and that removing this growth in a BR biosynthetic mutant of the aquatic plant *Utricularia gibba* (*Ugdwf4*) alters the internal structure of stems by mechanically constraining the expansion of faster-growing internal tissue (Kelly-Bellow et al., 2023). Previous work has also shown that epidermal expression of the BR receptor *BRI1* is sufficient to rescue leaf size in the loss-of-function *bri1-116* mutant background (Savaldi-Goldstein et al., 2007), so it is possible that BR acts in a similar way in both leaves and stems. As the epidermis has been proposed to drive intercellular air space expansion (Avery, 1933), and the BR synthesis mutant *Ugdwf4* in *U. gibba* has reduced intercellular air spaces in stems (Kelly-Bellow et al., 2023), we hypothesise that BR promotes epidermal growth in leaves to drive air space formation and patterning.

Here, we test this hypothesis by quantifying intercellular air spaces in the first leaf of *Arabidopsis thaliana* throughout development and analysing the effect of mutations in BR synthesis and perception. We show that loss of BR function does not affect air space formation, but causes air spaces to be gradually reduced in the palisade throughout leaf development. We further show that epidermal BR perception controls air space patterning non-cell autonomously, supporting a role for the epidermis in leaf air space development. Finally, we reduce epidermal growth by ectopic expression of the growth repressor *BIG BROTHER* (*BB*) (Disch et al., 2006), and show that this also reduces air space size. Overall, we propose that BR controls leaf air space patterning non-cell autonomously by promoting epidermal growth.

## RESULTS

### Leaf air spaces in the palisade and spongy mesophyll undergo different developmental trajectories in *Arabidopsis*

**Fig. 2.**
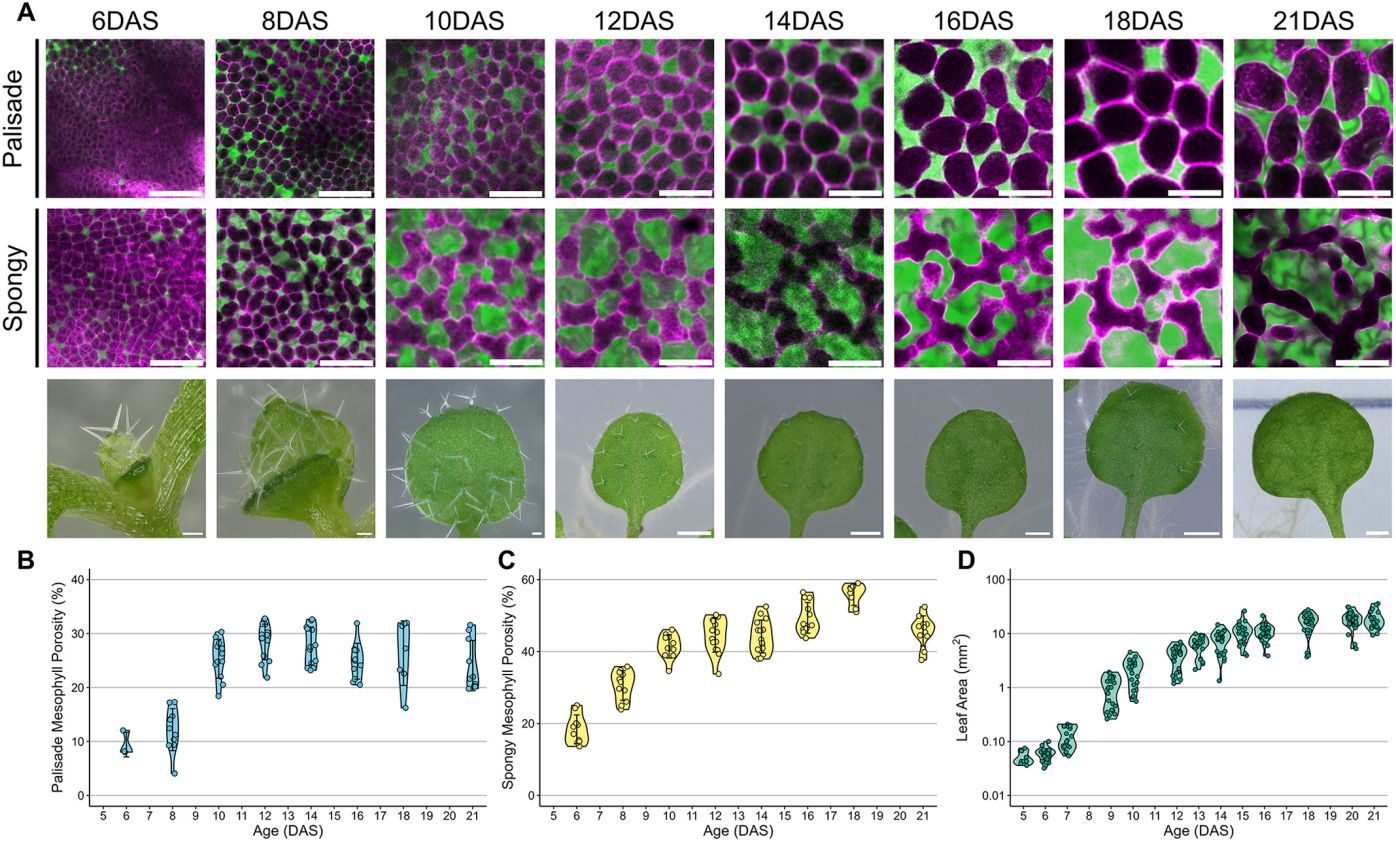
**Palisade and spongy mesophyll air spaces undergo different developmental trajectories in *A. thaliana*.** (A) Air spaces in the palisade (top row) and spongy (middle row) visualised by Nile Red dye. Air spaces are green, cell outlines magenta. Bottom row shows image of whole leaf for scale. (B,C) Palisade (B) and spongy (C) mesophyll tissue porosity (%) of leaf 1 over 21 days. (D) Leaf area of *A. thaliana* leaf 1 over the same time period. Data are mean±s.e.m. (6-8 DAS, *n*≥6; 10-21 DAS, *n*≥15). Scale bars: 50 µm (A, top two rows); 100 µm (A, bottom row, 6-10 DAS); 1000 µm (A, bottom row, 12-21 DAS).

To determine the developmental timeline for air space formation in leaves, we used Nile red dye to quantify porosity (percentage air space) of *A. thaliana* leaf one throughout development (Fig. 1; Kawase et al., 2015, 2016). Nile red emits two separate wavelengths of light when bound to a membrane or free in solution, allowing mesophyll cell membranes and intercellular air spaces to be visualised simultaneously (Fig. 2A). Leaf air spaces are first detectable by Nile red in the spongy and palisade mesophyll at 6 days after sowing (DAS) (Fig. 2A). As Nile Red dye marks air spaces by entering through stomata and stomata are not visibly open before 6 DAS we were unable to assess the porosity of leaves before 6 DAS using this method. To overcome this difficulty, we used the *pUBQ1::2x-tdTomato-29-1* line (Shapiro et al., 2015) to visualise mesophyll cell outlines at 4-6 DAS (Fig. S1). This showed that limited air spaces are present at 5 DAS, with no air spaces visible at 4 DAS in either spongy or palisade mesophyll.

As leaf development proceeds in a basipetal gradient (Pyke et al., 1991; Donnelly et al., 1999; Fox et al., 2018) we quantified leaf porosity at the midpoint of the leaf blade to better compare across timepoints (Fig. 2B,C). We observed markedly different trajectories for air space expansion in the palisade and spongy mesophyll. At 6 DAS, box shaped palisade mesophyll cells are mostly connected, with some small air spaces appearing at multicellular junctions (Fig. 2A,B; 10% porosity), whereas spongy mesophyll porosity is already 20% (Fig. 2C). From 6-21 DAS, spongy mesophyll porosity increased from 20% to 55%, with an initially faster rate that gradually slowed down until plateauing at ∼18 DAS. Over this same period, palisade mesophyll porosity increased more rapidly, from 10% at 8 DAS to ∼30% at 10 DAS, followed by no further increase despite leaf area continuing to expand (Fig. 2B,D). Together, these results highlight the different trajectories for air space development in the spongy and palisade mesophyll, and show that air space expansion mainly occurs between 8 DAS and 10 DAS in the palisade.

### Brassinosteroid signalling is required for air spaces in mature leaves

We have previously shown that BR promotes growth in the epidermis of stems (Kelly-Bellow et al., 2023), and epidermal growth has been proposed to drive air space expansion (Avery, 1933). Therefore, we hypothesised that BR may drive leaf air space expansion by promoting epidermal growth. To test if BR regulates air space patterning, we quantified tissue porosity in mature leaves of plants with reduced BR synthesis [*dwf4* mutants or plants treated with the BR synthesis inhibitor brassinazole (BRZ)] or perception (the BR receptor mutant *bri1-116*).

**Fig. 3.**
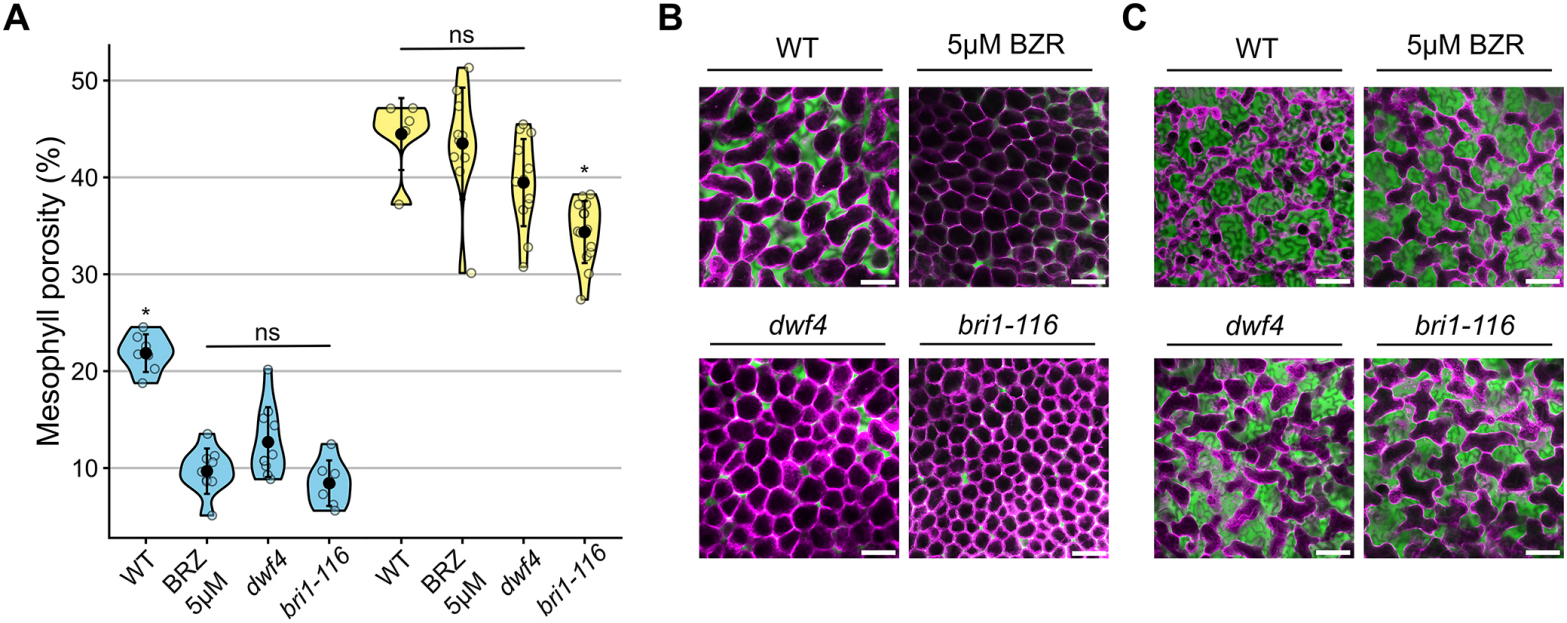
**Brassinosteroids are required for normal air space patterning in leaves.** (A) Porosity quantified using Nile Red dye from sub-epidermal palisade and spongy mesophyll tissue layers at 21 DAS. Blue is palisade mesophyll porosity and yellow is spongy mesophyll porosity. Plants were untreated wild type (WT), wild type plants grown in media containing 5 µM Brassinazole (BRZ), *dwf4* and *bri1-116* mutants. (B,C) Representative Nile Red stained palisade (B) and spongy (C) mesophyll images, with air spaces visible in green and cell outlines in magenta. *P<0.05, **P<0.01 (two-way ANOVA). ns, not significantly different. Data are mean±s.e.m. (*n*≥6). Scale bars: 50 µm.

In mature 3-week-old first leaves of *dwf4*, *bri1-116* and BRZ-treated plants, air spaces were significantly reduced in the palisade mesophyll from ∼25% to ∼10% (Fig. 3A,C; *P*<0.0002). Tissue porosity was also slightly but significantly reduced in the spongy mesophyll in *bri1-116* (Fig. 3A,B; from 45% to 35% porosity, *P*=0.0000153), whereas porosity in *dwf4* mutants and plants treated with 5 µM BRZ were not significantly different from wild type (Fig. 3A). These data suggest that BR is necessary for either air space initiation or expansion in the palisade mesophyll, with a minimal role in the spongy mesophyll.

### The BR perception mutant *bri1-116* forms air spaces in early leaf development which are lost by maturity

**Fig. 4.**
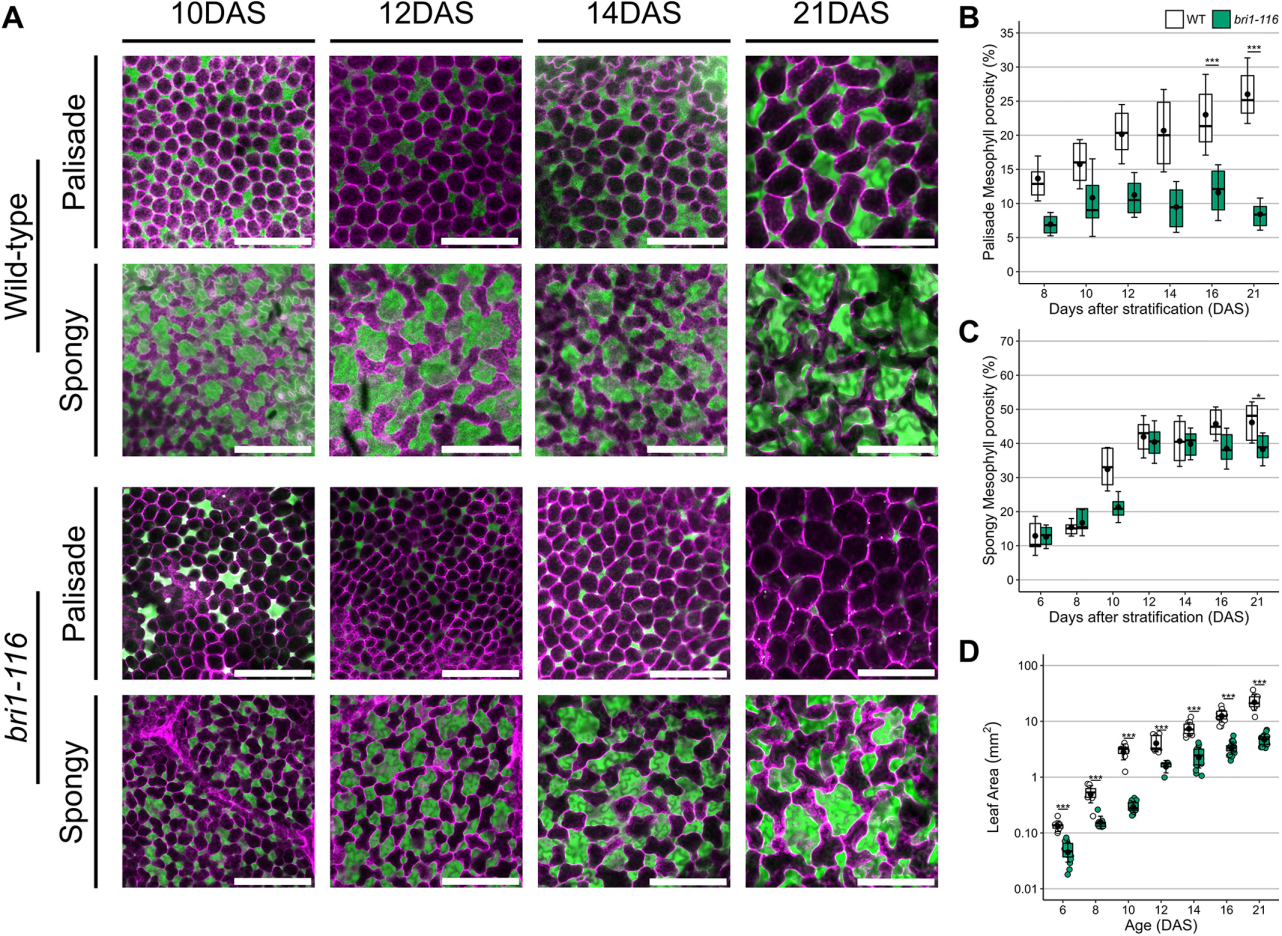
**Loss of BR perception reduces palisade air space expansion but does not affect initiation.** (A) Representative Nile Red stained spongy and palisade mesophyll images from Col-0 and *bri1-116* throughout the first 21 days of leaf development. Air spaces are visible in green and cell outlines in magenta. (B,C) Palisade (B) and spongy (C) mesophyll porosity quantified over the same time period. (D) Leaf area quantified over 21 days of leaf development. *P<0.05, ***P<0.001 (two-way ANOVA) (*n*≥20). Box plots show median values (middle bars) and first to third interquartile ranges (boxes); whiskers indicate 1.5× the interquartile ranges; dots indicate individual replicates. Scale bars: 100 µm.

To determine if air space formation or expansion is prevented by a lack of BR signalling, we analysed air space patterning and leaf area throughout leaf development in the *bri1-116* mutant by staining with Nile Red (Fig. 4A,D). As previously, palisade mesophyll air spaces were difficult to measure before 8 DAS. In young leaves at 8 DAS, palisade mesophyll porosity was not significantly different between wild type and *bri1-116* (*P*=0.9998917), suggesting that BR does not drive air space formation (Fig. 4B). From 8 DAS to 12 DAS, palisade porosity in both wild type and *bri1-116* increased from 10% to ∼20%. Following this, *bri1-116* palisade mesophyll air space gradually decreased until ∼7% at 21 DAS, whereas wild-type palisade porosity increased to ∼25%. Spongy mesophyll porosity increased similarly in both wild type and *bri1-116* to ∼40% at 12 DAS (*P*=0.9990888) (Fig. 4A,C). After this point, wild-type spongy porosity continued to increase to ∼45% at 21 DAS, whereas *bri1-116* plateaued, ending up slightly, but significantly, smaller than wild type at 21 DAS (Fig. 4C; *P*=0.0362756). Spongy mesophyll cell morphology also appeared to be unaffected in *bri1-116* (Fig. 4A). These data show that lack of BR perception does not affect air space initiation, but strongly reduces air space expansion and maintenance in the palisade, but not the spongy, mesophyll.

### Epidermal brassinosteroid signalling is sufficient for air space expansion

As palisade air spaces get smaller throughout development in the *bri1-116* mutant, but not in wild type, the growth rate of palisade tissue must be relatively higher compared to the epidermis in *bri1-116* compared to wild type. This could be due to either higher growth in the palisade or lower growth in the epidermis.

One hypothesis to explain the gradual reduction of air space in the *bri1-116* mutant is that, in wild-type plants, BR promotes epidermal growth, allowing internal tissue to grow with enough room for intercellular spaces to expand. Under this hypothesis, the *bri1-116* mutant exhibits reduced epidermal growth, but maintains normal mesophyll growth. This epidermal growth restriction constricts the expanding mesophyll tissue and presses palisade mesophyll cells together, resulting in a reduction of air space throughout development. This hypothesis is supported by the strongly reduced leaf size in *bri1-116*, which highlights a reduction in epidermal growth.

**Fig. 5.**
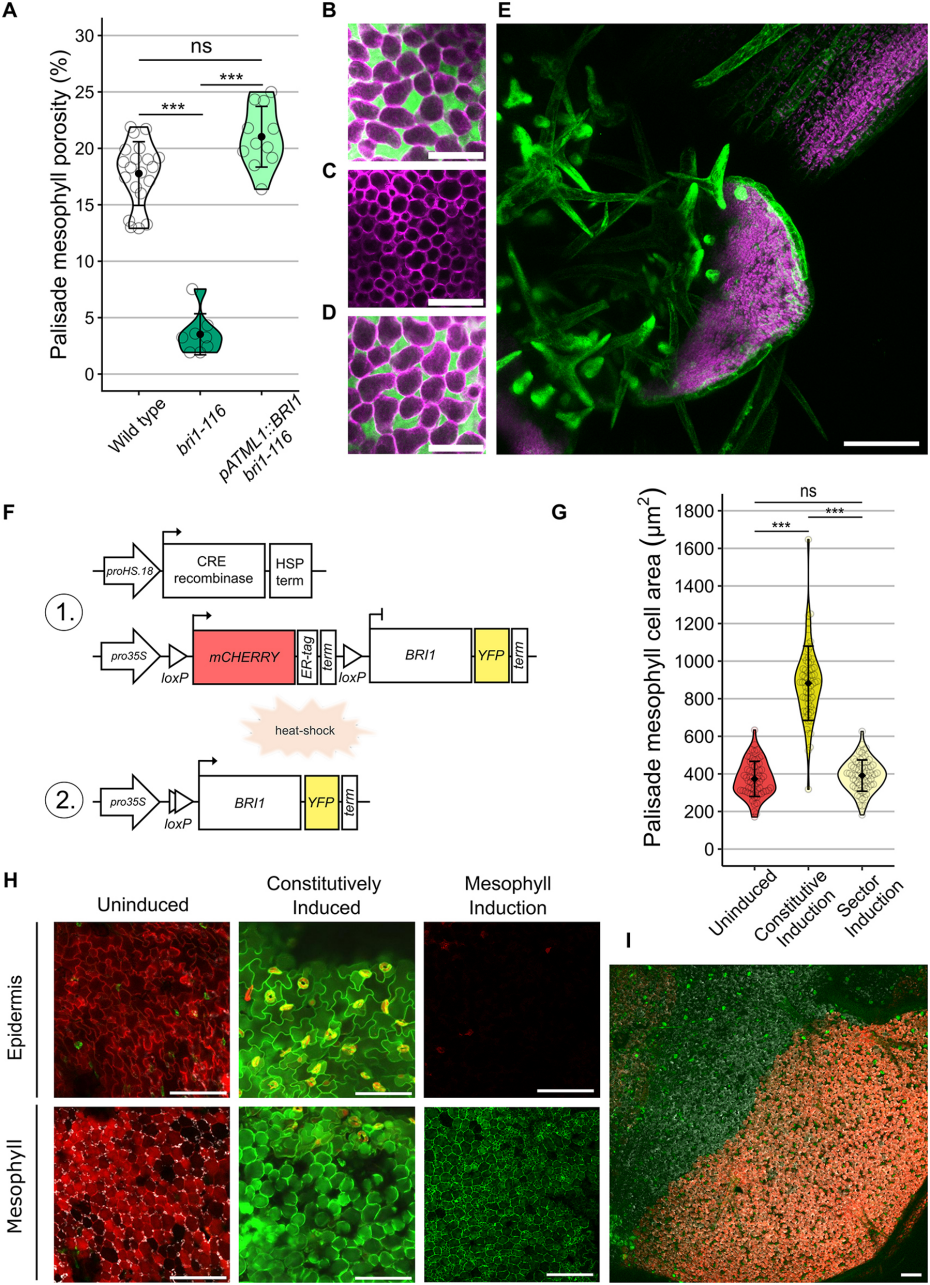
**Epidermal, but not mesophyll, expression of *BRI1* is sufficient to rescue mesophyll air space.** (A) Quantification of palisade mesophyll porosity in leaf 1 and leaf 2 of wild type, *bri1-116* mutants and *bri1-116* mutants rescued with epidermal *BRI1-GFP* at 21 DAS. (B-D) Palisade mesophyll of wild type (B), *bri1-116* (C) and *bri1-116 pATML1::BRI1* rescue (D) leaves imaged with Nile Red dye. (E) Leaf 2 of a *bri1-116 p35S::BRI1-GFP* rescue at 7 DAS. Air spaces are visible in green and cell outlines in magenta. (F) Diagram of the heat-shock inducible CRE-lox recombinase system used to rescue *bri1-116* mutants with *p35S::BRI1-VENUS*. (G) Palisade mesophyll cell size of *bri1-116* mutants is rescued by constitutively induced *p35S::BRI1*, but not in sectors. (H) Representative images of uninduced, constitutive induction and mesophyll only *p35S::BRI1-VENUS*. mCHERRY expressed in uninduced cells can be seen in red, BRI1-VENUS expressed in rescued cells can be seen in green. (I) Representative image of 21 day old leaf induced at 3 days old showing both YFP and RFP sectors. *P<0.05, ***P<0.001 (Kruskal–Wallis rank sums test). ns, not significantly different. Data are mean±s.e.m. (*n*≥10 in A; cells from ≥4 leaves in G). Scale bars: 100 µm.

If the above hypothesis is correct, BR signalling in the epidermis should be sufficient to drive air space expansion in the palisade mesophyll. To determine if this is the case, we quantified the porosity of *bri1-116* mutants transformed with a *BRI1* tissue specific epidermal rescue line, *pATML1::BRI1-GFP* (Savaldi-Goldstein et al., 2007). As previously described, the epidermal rescue of *BRI1* signalling restored leaf shape and size and closely resembled a wild-type plant rosette (Fig. S2). Additionally, epidermal rescue of *BRI1* function rescued palisade mesophyll porosity to wild type-levels (Fig. 5A-E; *P*=0.0795873). Overall, this suggests that BR signalling via the epidermis non-cell autonomously enables the retention of air spaces in the palisade mesophyll.

### Mesophyll-specific brassinosteroid signalling does not rescue palisade cell size or air space patterning

Recent work has shown that *BRI1* contains regulatory elements within its coding sequence that can drive low levels of constitutive expression (Blanco-Touriñán et al., 2024). Therefore, an alternative explanation of the above data is that low levels of BRI1 in the mesophyll of *AtML1::BRI1* act cell autonomously to rescue palisade patterning. If this is the case, then mesophyll-specific expression of *BRI1* should rescue palisade cell morphology and air space patterning. To test this hypothesis, we generated a version of BRI1-GFP that can be induced in clonal sectors via heat shock (HS)-inducible CRE-mediated lox site recombination in the *bri1-116* mutant (Fig. 5F; Kuchen et al., 2012; Lee et al., 2019). Uninduced plants express constitutive RFP and are phenotypic *bri1-116* mutants (Fig. 5F-H), whereas constitutive induction at 3 DAS rescues overall leaf size (Fig. 5F-H). Mesophyll-specific clonal sectors induced at 3 DAS express *BRI1-VENUS*, but palisade cells remain tightly packed and cell size is not rescued (Fig. 5G-I). These data show that mesophyll-specific expression of *BRI1* is not sufficient for normal palisade expansion or air space patterning. Together with the epidermal rescue experiments above, it is likely that BRI1 acts predominantly in the epidermis to drive air space patterning non-cell autonomously.

### Mesophyll cell size is reduced, but division rate unaltered in *bri1-116*

**Fig. 6.**
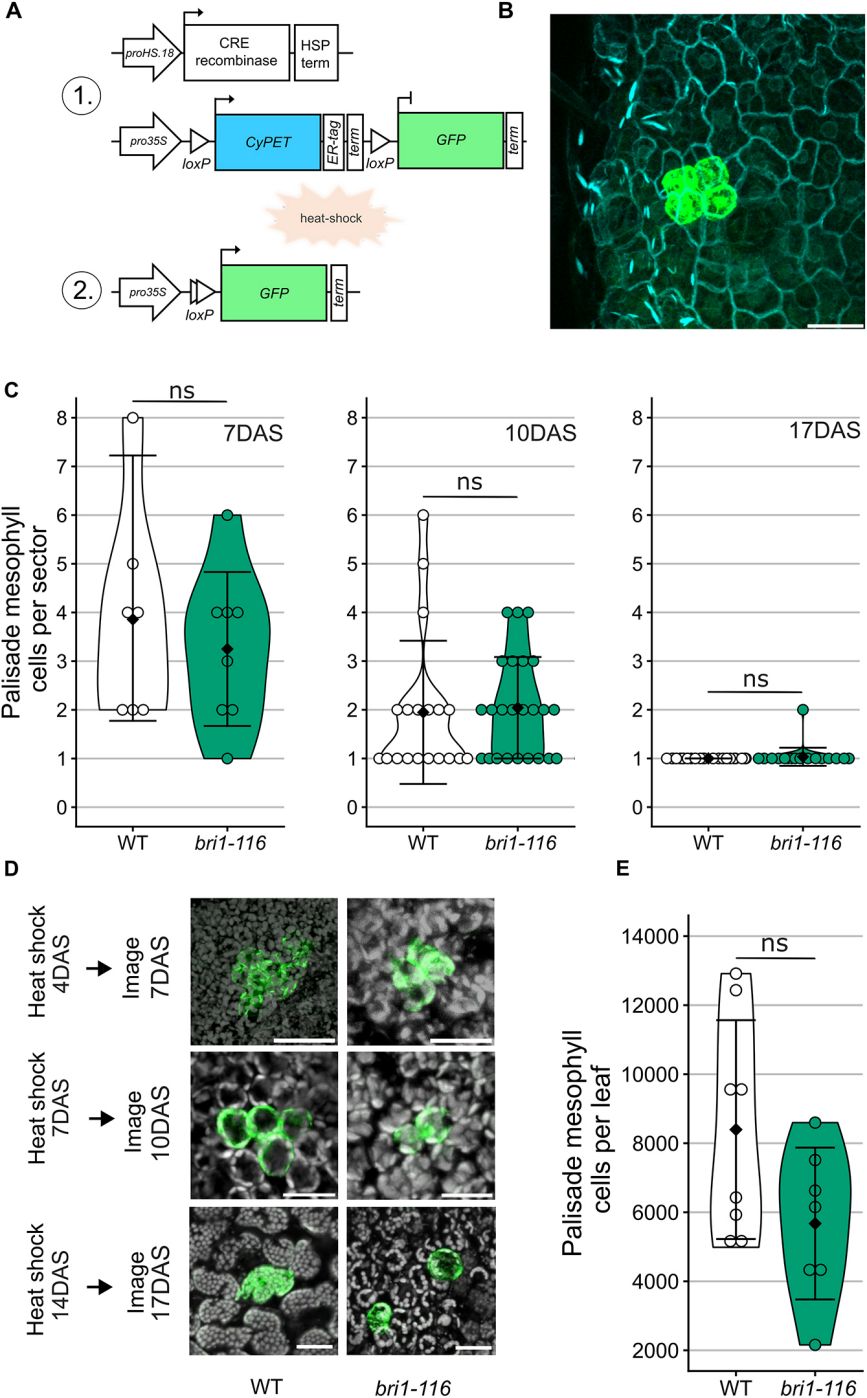
**Palisade mesophyll cell division is not increased in BR signalling mutants.** (A) Diagram of the heat-shock inducible CRE-lox recombinase system. (B) Representative sector illustrating the CyPET background and GFP in induced mesophyll cells. (C) Quantification of cells in sectors expressing GFP in palisade mesophyll of 7 DAS leaves induced at 4 DAS, 10 DAS leaves induced by heat-shock at 8 DAS, and 17 DAS leaves induced at 14 DAS. (D) Wild-type and *bri1-116* palisade mesophyll sector expressing GFP in a 7 DAS leaf 3 days after sector induction, a 10 DAS leaf 2 days after sector induction, and a 17 DAS leaf 3 days after sector induction. Chlorophyll autofluorescence visualised in grey. (E) Quantification of the number of palisade mesophyll cells in wild-type and *bri1-116* mutant leaf 1 and leaf 2 at 21 DAS by extrapolation based on leaf area. *P<0.05 (unpaired two-tailed Student's *t*-test). ns, not significantly different. Data are mean±s.e.m. (*n*≥10 sectors from different leaves). Scale bars: 20 µm.

An alternative hypothesis to explain the reduced air space and increased cell density of the *bri1-116* mutant is that growth in the mesophyll (cell division or expansion) is increased, meaning that more cells or larger cells are squashed into the same area. To test whether mesophyll cell division was increased in *bri1-116*, we calculated palisade cell number per leaf in both wild type and *bri1-116* (Fig. 6E). This showed no significant difference between wild type and *bri1-116*, despite *bri1-116* leaves being significantly smaller (*P*>0.05). This suggests that division rates are broadly similar. To better quantify whether cell division is increased in *bri1-116* throughout development, we performed clonal analysis by using HS-inducible CRE-mediated lox site recombination to induce GFP expression (Fig. 6A,B) (Kuchen et al., 2012; Lee et al., 2019). We induced GFP expression in single cells and counted the number of cells in each clone 2-3 days later as a proxy for cell division rate. These data show that cell number per clone was not significantly different between wild type and *bri1-116* at three different timepoints across palisade development (4-7, 8-10 and 14-17 days, *P*>0.05) (Fig. 6C,D).

To test whether reduced palisade air spaces are due to increased mesophyll cell expansion in *bri1-116*, we quantified palisade mesophyll cell size in Col-0 and *bri1-116.* This showed that palisade cells in *bri1-116* were smaller than in wild type, suggesting that increased cell expansion does not drive air space reduction (Fig. S4C). Quantification of epidermal cell size showed concomitant decreases, while stomatal density was increased (Fig. S4A,B). These data are in line with previous quantifications of cell size in *bri1-116* (Zhiponova et al., 2013) and highlight that BR affects air spaces independently of stomata. Overall, these data suggest that the decrease in palisade tissue porosity and increase in cell density observed in *bri1-116* is likely due to a restriction in epidermal growth while division rate in the palisade is maintained.

### Decreased epidermal growth via tissue-specific expression of *BIG BROTHER* decreases mesophyll air space

**Fig. 7.**
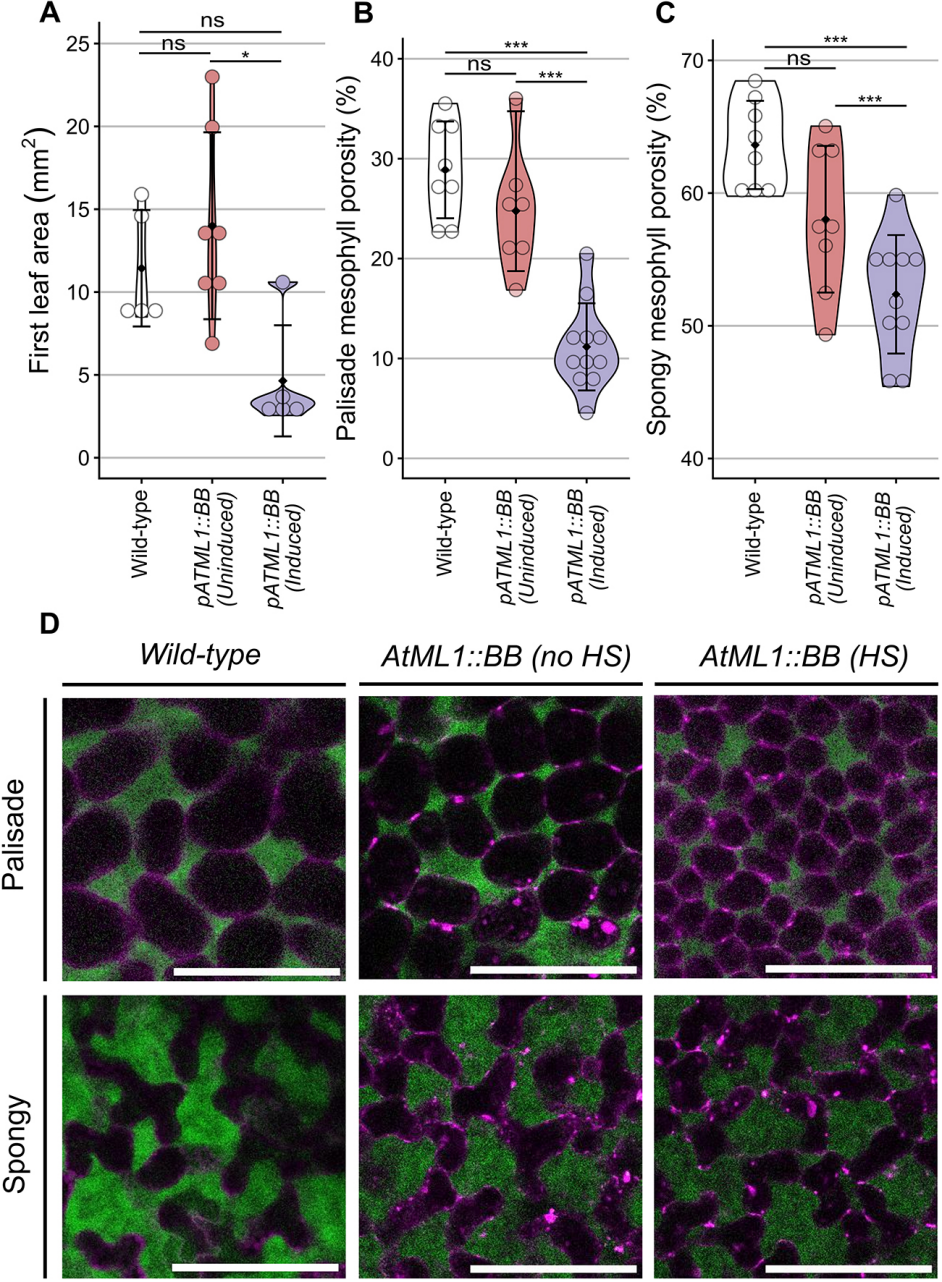
**Repression of epidermal growth reduces mesophyll porosity.** (A) Induction of epidermal *BB* at 3 DAS reduces leaf 1 area at 21 DAS. (B-D) Epidermal *BB* induction reduces palisade mesophyll porosity (B,D) but not spongy mesophyll porosity (C,D). Air spaces are visible in green and cell outlines in magenta. HS, heat shock. *P<0.05, ***P<0.001 (one-way ANOVA). ns, not significantly different. Data are mean±s.e.m. (leaf area *n*≥5, porosity *n*≥8). Scale bars: 100 µm.

If the reduced porosity of palisade tissue of BR mutants is due to reduced epidermal growth constraining the expansion of inner tissue, it should be possible to reduce palisade mesophyll porosity by reducing growth in the epidermis independently of BR. To do this we used HS-inducible CRE-mediated lox site recombination to induce expression of the negative growth regulator *BB* (Disch et al., 2006) under control of the *AtML1* promoter to reduce growth specifically in the epidermis (Fig. S3). BB is an E3 ubiquitin ligase that degrades positive regulators of leaf growth and restricts the duration of proliferative growth (Disch et al., 2006). To prevent intercellular movement and ensure epidermal-specific expression we tagged BB with three VENUS fluorescent proteins. We induced epidermal BB expression at 3 DAS (before air spaces form) and quantified leaf size and porosity at 21 DAS, when leaves were fully expanded. Leaves in which epidermal *BB* expression was induced were reduced in area compared to uninduced and wild-type controls (Fig. 7A) and had significantly lower palisade porosity than wild type; 10% in induced *AtML1::BB* versus 25% in wild type (Fig. 7B,D; P≤0.04). Spongy palisade porosity was also reduced in induced *AtML1::BB* leaves, but more moderately; 50% in induced AtML1::BB versus 60% in wild type (Fig. 7C,D). In line with *bri1-116*, palisade and epidermal cell size is also reduced in *AtML1::BB*, and stomatal density is increased (Fig. S4). These data suggest that reducing epidermal growth may reduce air space expansion in the mesophyll tissue non-cell autonomously, independently of stomata.

## DISCUSSION

**Fig. 8.**
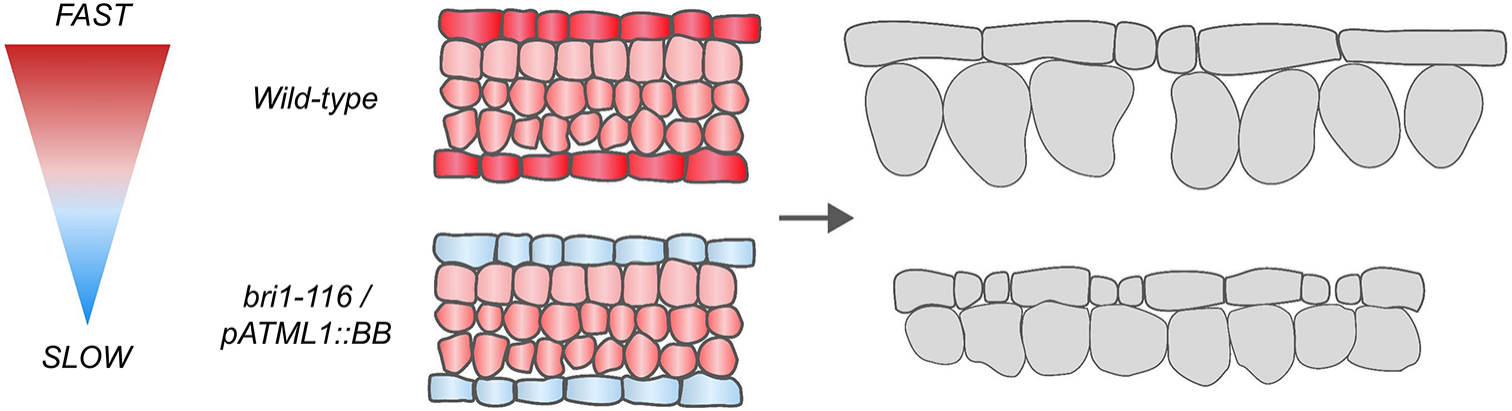
**Reduction in epidermal growth constrains air space expansion in the palisade mesophyll.** In wild-type leaves, the epidermis grows slightly faster than the mesophyll, allowing air spaces to expand. In *bri1-116* and *AtML::BB lines*, epidermal growth is reduced, but mesophyll growth is unaffected, causing air spaces to be removed as cells press against each other.

Here, we show that BR signalling in the epidermis is necessary to pattern intercellular air spaces in leaf mesophyll tissue. Specifically, we show that BR is not required for air space initiation, but instead is required for retention and expansion of air spaces in the palisade mesophyll as the leaf expands. By experimentally reducing growth rate in the epidermis by epidermal-specific expression of the negative growth regulator *BB*, we also demonstrate that restricting epidermal growth is sufficient to reduce palisade mesophyll porosity. This is consistent with previous work showing that limiting cell division in the leaf epidermis leads to reduced palisade mesophyll porosity, but not spongy mesophyll porosity (Lehmeier et al., 2017). Therefore, we propose that BR drives epidermal growth to determine the pattern of intercellular air spaces in the palisade mesophyll non-cell autonomously (Fig. 8).

### Epidermal growth determines leaf air space pattering

Our data suggest that epidermal growth is necessary to allow intercellular air spaces to expand in the palisade mesophyll throughout leaf development. Previously, the epidermis has been proposed to drive air space formation and expansion by growing faster than the mesophyll cells and physically pulling them apart (Avery, 1933). Our data suggest that this is unlikely to be the mechanism driving initial air space formation (as lines with reduced epidermal growth still initiate air spaces), but it is possible that a faster growing epidermis acts to enhance air space expansion later in leaf development by applying tension to slower growing inner layers. Alternatively, epidermal growth may not drive air space expansion, but might simply need to be high enough not to mechanically constrain the mesophyll tissue as it grows. These two hypotheses may be able to be distinguished in the future by further experiments manipulating growth rates specifically in the mesophyll and epidermis, or by analysing periclinal chimeras of plants with differing growth patterns (as in Marcotrigiano, 2010).

These data contrast with the prevailing model of stem elongation, where faster growing internal tissue drives growth by putting a slower growing, stiffer, epidermis under tension. Instead, in the leaf the epidermis must grow sufficiently to allow (or drive, depending on the hypothesis) appropriate patterning of internal tissues.

### Non-cell autonomous effects of BR signalling

Our work here suggests that the non-cell autonomous effects of BR signalling in air space patterning are not driven by intercellular molecular signalling, but instead by the mechanical constraints of neighbouring tissues growing at different rates (Coen and Rebocho, 2016). This process may also underlie the non-cell autonomous effects of BR in other tissues (e.g. Vragović et al., 2015). Although the role of BR in the epidermis appears to be dominant in air space patterning, it is also possible that BR acts directly in mesophyll cells to control other traits, as has recently been shown in rice bundle sheath cells, where BR regulates chloroplast area (Cackett et al., 2025). Future work with mesophyll-specific BRI1 rescue lines can test this hypothesis.

In addition, our data quantify air space patterning and cell size in two dimensions in the plane of the leaf. As BR has previously been shown to alter cell shape in 3D rather than overall cell volume in roots (Fridman et al., 2021), it is possible that, in BR mutants, reduction of growth in the plane of the leaf is compensated by increased growth in thickness. As air spaces expand primarily in the plane of the leaf this difference would not affect our conclusions, but it may provide an alternative basis for the differences in growth we observe.

### Different developmental mechanisms control air space patterning in the spongy and palisade mesophyll

Previous studies have described early spongy mesophyll development in detail (Zhang et al., 2021; Zhang and Ambrose, 2022), illustrating how air spaces first appear at multi-cell junctions after the degradation of the middle lamella, followed by intracellular turgor and microtubule patterning causing spongy mesophyll cells to change their shape from box-like to rounded to lobed. Consistent with previous studies, our results indicate that both in the spongy and the palisade layers, air space initiation occurs at multicellular junctions (Sifton, 1945; Fox et al., 2018). However, our work shows that air space formation and expansion follow different trajectories in each mesophyll cell type: air spaces expand gradually throughout leaf development in the spongy, whereas the majority of air space expansion in palisade air space occurs between 8 DAS and 10 DAS (Fig. 2). The period of high air space expansion in the palisade is coincident with the highest rate of leaf expansion, consistent with epidermal expansion having the largest effect on air spaces in this tissue layer.

Both BR and epidermal *BB* expression also have a stronger effect on palisade air space development compared to spongy: palisade porosity in *bri1-116* strongly decreased from 12 DAS onwards, while spongy porosity was less affected (Fig. 4), and palisade porosity in AtML::BB is halved, whereas spongy porosity is reduced by ∼10%. Mature spongy mesophyll cells in both of these lines also form complex lobed shapes similar to wild type (Figs 3C, 4A and 7). Together these data suggest that spongy mesophyll cell morphogenesis is not strongly affected by epidermal growth and is controlled cell autonomously, independently of BR. Recent work has shown that microtubule orientation controls spongy mesophyll morphogenesis (Zhang and Ambrose, 2022; Zhang et al., 2021), and misexpression of Rho of Plant 2 (ROP2) and ROP-interactive CRIB-containing protein 1 (RIC1) also disturbs spongy mesophyll morphology (Østerlund et al., 2025). Overall, it seems likely that a cell autonomous microtubule-driven mechanism determines spongy mesophyll morphogenesis, whereas palisade tissue patterning is more influenced by organ-wide factors. However, it is possible that the greater reduction of palisade air space in both *bri1-116* and *AtML1::BB* is due to a stronger reduction of growth in the upper epidermis, causing a stronger effect on air spaces in the palisade. This seems unlikely as *bri1-116* and *AtML1::BB* leaves do not curve upwards as would be expected if the upper epidermis grew slower (Coen and Rebocho, 2016), but remains to be tested.

### Interaction of BR with known mechanisms of air space patterning

It is currently unclear how the non-cell autonomous role of BR interacts with known regulators of air space patterning: stomata, chloroplasts and the microtubule cytoskeleton (Whitewoods, 2021).

It is clear that BR represses stomatal formation, and the *bri1-116* mutant has increased stomatal density (Kim et al., 2012; Fig. S4A). As stomata are associated with sub stomatal air chambers, they are generally positively correlated with palisade porosity (Lundgren et al., 2019). Therefore, the decreased porosity of the *bri1-116* mutant alongside increased stomatal density suggests that BR is necessary for stomata to promote air space formation. We hypothesise that epidermal growth acts in parallel to a stomatal signal and is necessary to allow substomatal cavities to expand. This is in line with our observation that the *AtML1::BB* line also has reduced palisade air spaces without a concomitant decrease in stomatal density (Fig. S4A). However, it is also possible that stomata signal via BR to locally promote air space formation.

Several mutants with defects in chloroplast biogenesis or metabolism are also known to have more extensive leaf air spaces (Li et al., 1995; González-Bayón et al., 2006). How BR interacts with chloroplasts to control air space patterning is currently unknown. We hypothesise that they are parallel pathways that regulate air space patterning independently. Double mutants or inhibiting BR synthesis chemically in chloroplast mutants will be necessary to test this.

Evidence from several studies (Zhang and Ambrose, 2022; Zhang et al., 2021) suggests that local reorientation of microtubules towards air space-adjacent cell walls controls spongy mesophyll lobe formation and air space patterning. As discussed above, lobe formation in spongy mesophyll cells appears to be unaffected in BR mutants. This is consistent with computational models of spongy mesophyll morphogenesis, which show that when tissue growth is constrained by an external force, spongy mesophyll cell lobe formation is unaffected but tissue porosity reduces (Treado et al., 2022). Together, these data point to a model where cell autonomous and non-cell autonomous mechanisms work in parallel to generate the overall pattern of air space in a leaf.

### Conclusions

Overall, we show that BR controls leaf air space patterning non-cell autonomously by promoting epidermal growth (Fig. 8). It remains to be seen whether this mechanism is simply a necessary prerequisite for air space expansion, or whether plants actively modulate epidermal BR signalling to alter air space patterning in response to environmental cues.

## MATERIALS AND METHODS

### Plant material and growth conditions

Plants were grown on plates containing MS media [0.441% Murashige and Skoog including vitamins, 0.05% (w/v) MES, 0.8% Difco agar, pH to 5.7]. Sterilised seeds were stratified in the dark at 4°C for 2 days before growing in a controlled environment chamber at 20°C with 16 h of illumination (150 μmol m^−2^ s^−1^).

BRZ (Sigma-Aldrich) was dissolved in water and added to plates to a final concentration of 5 µM.

### Freeze-fracture SEM

A mature *Arabidopsis* leaf 1 (4 weeks old) was stuck vertically on a brass stub with a cryo glue preparation consisting of a 3:1 mixture of Tissue-Tec (Scigen Scientific) and Aquadag colloidal graphite (Agar Scientific). It was plunge frozen in liquid nitrogen with vacuum applied and fractured using a level semi-rotary cryo knife. cryogenic scanning electron microscopy (Cryo-SEM) imaging was carried out using a Zeiss EVO HD15 SEM fitted with a cryo-stage as described in Wightman et al. (2017) with the following modifications: (1) Iridium 169 was used as the sputter coating target to a measured thickness of 3 nm; and (2) imaging used the backscattered electron 170 detector and a gun voltage of 25 kV and a probe current of 16 pA.

### Nile Red staining and confocal imaging

Nile Red dye (Sigma-Aldrich) was dissolved in 1.0 cs Silicon oil (Aston Chemicals) by shaking overnight to a final concentration of 10 μM. Leaves were stained by soaking in Nile Red solution for 10 min before mounting on a slide under a coverslip and imaging on a Leica SP8 confocal microscope using a 20× dry objective. Nile Red bound to cell membranes was excited with a 561 nm wavelength laser and visualised between 565 and 640 nm, and Nile Red free in solution was excited with a 488 nm wavelength laser and visualised between 490 and 560 nm. To quantify leaf porosity (percentage air space in a region of tissue) unbound Nile Red fluorescence was quantified by automatic thresholding in ImageJ as described previously (Kawase et al., 2015, 2016), and porosity was defined as the percentage area of a 2D region that contained air space. To accurately identify palisade and spongy mesophyll tissue throughout development before defining morphological characteristics emerged, palisade and spongy mesophyll tissue were defined by position: palisade as the most adaxial layer of subepidermal mesophyll cells and spongy as the most abaxial layer of subepidermal mesophyll cells. When imaging BRI1-VENUS in the BRI1 sector line, Leica Tau-Gating was used to separate chlorophyll autofluorescence from the VENUS tag based on fluorescence lifetime.

### Leaf area and cell counts

Whole plants were imaged using a Keyence VHX8000 microscope, and leaf area quantified using ImageJ. Cell counts per leaf were calculated by manually counting palisade cell number in a 200×200 μm region of palisade tissue from a leaf stained with Nile Red. Cell number per leaf was calculated by extrapolating the cell number per unit area to the whole leaf area for each genotype.

### Construct generation

The GFP sector line contains two constructs: a p35S-loxPrev-CyPET-t35S-loxPrev-GFP-tAct sector construct (EC71028) and a pHSP18.2::CRE-tAtHS construct (EC71692). These were both generated by Golden Gate cloning as previously described (Weber et al., 2011): see Genbank files EC71028 and EC71692 (doi.org/10.6084/m9.figshare.32090665) for final construct sequences, and Table S1 for Golden Gate module details. Plants were transformed with both constructs by floral dip and selected on plates using Kanamycin and Hygromycin selection. Homozygous T3 lines were crossed to *bri1-116* heterozygotes, and F2 offspring homozygous for *bri1-116* and containing the sector constructs were used for sector analysis.

The epidermal inducible BIG BROTHER line (AtML::BB) contains a pL2B-BAR-HS18.2:CRE-U5-Cre-pAtML1::loxP-CyPET-t35S-loxP-BB-3xVenus-tAct (CW0091). CW0091 was also generated by Golden Gate cloning. A triple Venus tag was used to prevent intercellular movement and restrict BB to the epidermis (as previously described by Kawade et al., 2013). See Genbank file CW0091 (doi.org/10.6084/m9.figshare.32090665) for final construct sequence and Table S1 for Golden Gate module details.

The inducible BRI1 sector line contains pL2B-BAR-HS18.2:CRE-U5-Cre-p35S::loxP-CyPET-t35S-loxP-BRI1-GFP-tAct (CW0153). CW0153 was also generated by Golden Gate cloning. See Genbank file CW0153 (doi.org/10.6084/m9.figshare.32090665) for the annotated sequence and Table S1 for Golden Gate module details.

### Sector analysis and heat shock induction

CRE expression is temporarily induced by heat shocks of 39°C. The length of heat shock can be used to vary the extent of induction: short heat shocks of around 1-2 min cause CRE expression in very few cells and generate clonal sectors of induction, whereas longer heat shocks of 12 min cause widespread CRE expression and generate constitutive induction. We use each of these approaches to answer different questions.

Plants of the GFP sector line were grown on plates and heat shocked by floating in a 39°C water bath for 1-2 min. Heat-shocked plants were returned to the growth room and left to grow for 2 or 3 days before imaging GFP clones in leaf 1 using a Leica SP8 confocal microscope. Cell numbers per clone were manually counted in multiple sectors across five leaves.

Plants of the AtML1:BB line were grown on plates and heat shocked at 3 days old by floating in a 39°C water bath for 12 min to induce constitutive CRE recombination and ectopic *BB* expression in the entire epidermis.

Plants of the *35S::BRI1-VENUS* line were grown on plates and heat shocked at 3 days old by floating in a 39°C water bath. They were heat shocked for either 12 min to induce constitutive CRE recombination and *BRI1* expression, or for 1-2 min to induce clonal sectors of *BRI1*. Although constitutive induction rescued leaf size, BRI1-GFP fluorescence was dim and often only visible at 20× magnification with tau gating. Therefore, clonal sectors were identified by absence of RFP fluorescence.

### Statistical and image analyses and figure design

Statistical analyses and graph plotting were performed in R. Cell segmentation was performed using BioVoxxel, or manually in ImageJ by tracing cell outlines. Quantification of cell size was performed in ImageJ using the analyse particles command. Sample sizes are listed in the figure legends. ANOVA with Tukey HSD was used for statistical analysis unless otherwise listed in the figure legend. Statistical significance is denoted as: *P<0.05, **P<0.01, ***P<0.001.

## Supplementary Material



10.1242/develop.205110_sup1 Supplementary information

Table S1. GoldenGate parts used for cloning
