# Supplementary material for: Brassinosteroid controls leaf air space patterning non-cell autonomously by promoting epidermal growth
Source: Development. 2026 May 26;153(16):dev205110. doi: 10.1242/dev.205110 (PMC13286358; doi:10.1242/dev.205110)
Supplement: Supplementary information [file develop-153-205110-s1.pdf]

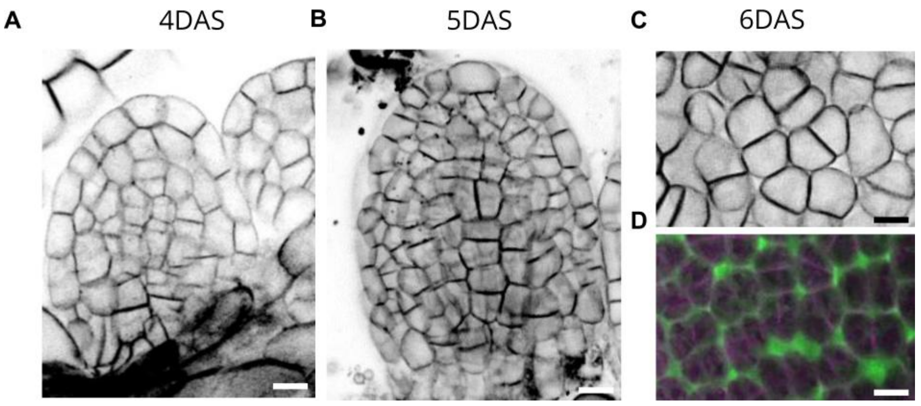

Fig. S1. Air space formation happens around 5 DAS. (A-C) Confocal images of pUBQ1::2x-tdTomato- 29-1 leaves from 4 – 6 DAS. (A) 4 DAS (B) 5 DAS and (C) 6 DAS. (D) Nile red dye staining of the abaxial side of leaf 2 at 6DAS. Scale bars = 10µm.

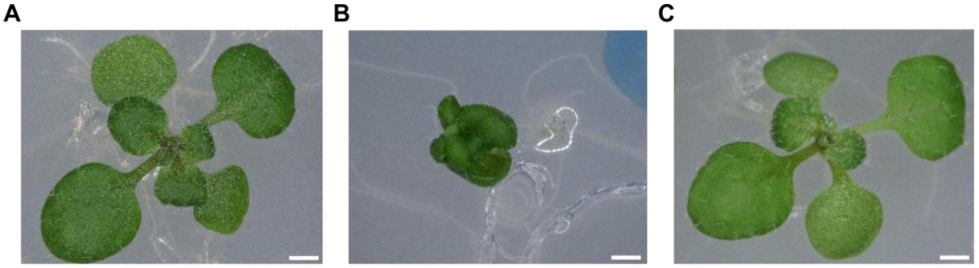

Fig. S2. epidermal expression of *BRI1* rescues leaf size in *bri1-116*. (A-C) 14-day old *A. thaliana* seedlings; WT (A), *bri1-116* (B) or (C) *bri1-116 pATML1::BRI1-GFP*. Scale bars = 1mm.

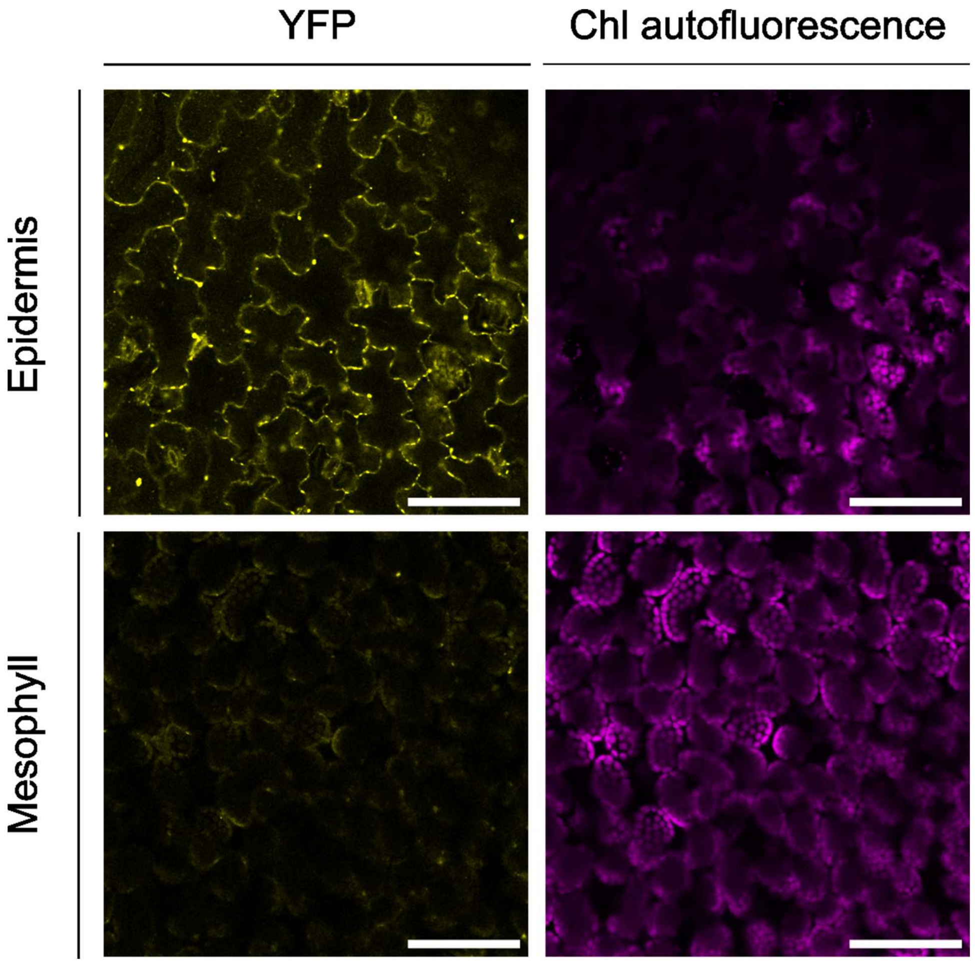

Fig. S3. *AtML1::BB-3xVENUS* is induced specifically in the epidermis. Confocal image of a heat shock-induced 21 day old leaf where *BB-3xVENUS* expression was induced at 3 DAS. Palisade mesophyll cells lack VENUS expression, whereas epidermal cells show bright VENUS. VENUS fluorescence is yellow and Chlorophyll autofluorescence magenta. Scale bars = 100µm.

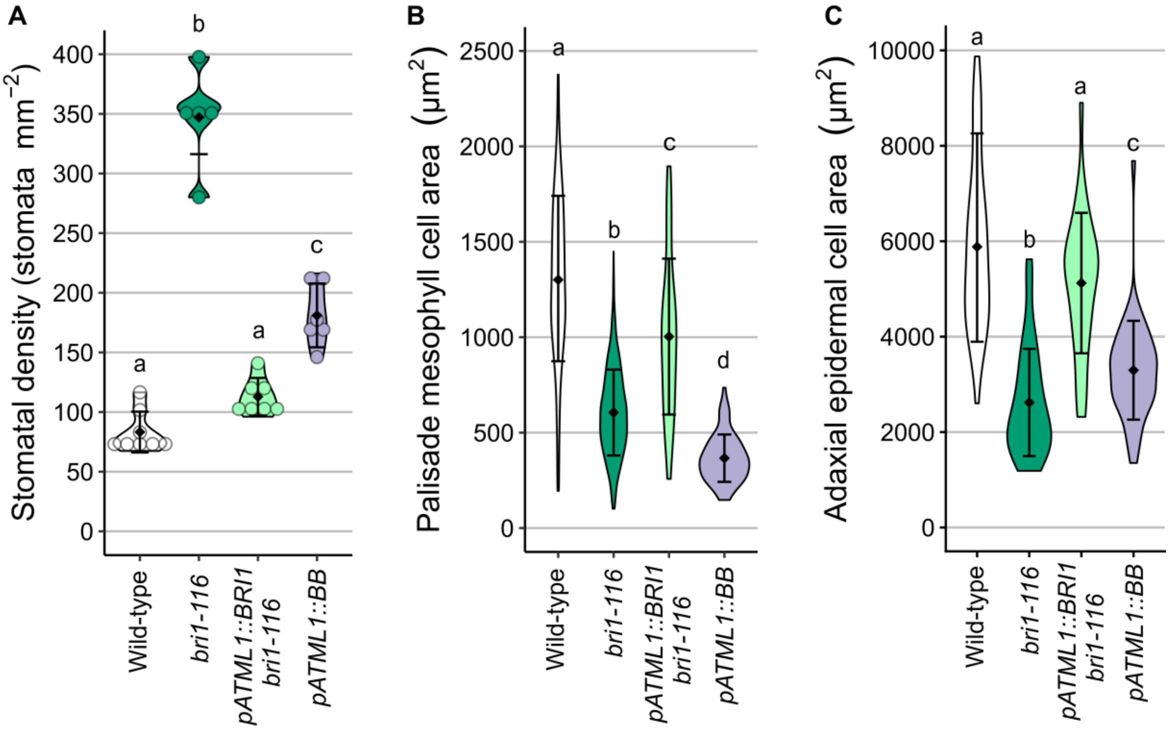

Fig. S4. Cell size is reduced, and stomatal density increased, in *bri1-116* and *AtML1::BB*. Quantification of stomatal density (A), palisade mesophyll cell area (B) and adaxial epidermal cell area (C) of 21 day old leaves. (Stomatal density n ≥ 5 leaves, cell area ≥ 400 cells from n ≥ 5 leaves).

Table S1. GoldenGate parts used for cloning

Available for download at
https://journals.biologists.com/dev/article-lookup/doi/10.1242/dev.205110#supplementary-data

Development • Supplementary information
