# Supplementary material for: Brassinosteroid controls leaf air space patterning non-cell autonomously by promoting epidermal growth
Source: Development. 2026 May 26;153(16):dev205110. doi: 10.1242/dev.205110 (PMC13286358; doi:10.1242/dev.205110)
Supplement: Supplementary file 1 [file develop-153-205110_review_history.pdf]

Development

Brassinosteroid controls leaf air space patterning non-cell autonomously by promoting epidermal growth

James M. Fitzsimons, Ana B. Rock, Richard L. De Falbe, Samantha Fox and Chris D. Whitewoods

DOI: 10.1242/dev.205110

Editor: Dominique Bergmann

Review timeline

Original submission:	18 July 2025
Editorial decision:	15 August 2025
First revision received:	2 February 2026
Editorial decision:	22 February 2026
Second revision received:	18 March 2026
Accepted:	18 March 2026

Original submission

First decision letter

MS ID#: dev.205110

MS TITLE: Brassinosteroid controls leaf air space patterning non-cell autonomously by promoting epidermal growth

AUTHORS: James M. Fitzsimons, Ana B. Rock, Richard L. De Falbe, Samantha Fox and Chris D. Whitewoods

Dear Dr Whitewoods (Dear Chris),

I have now received all the referees' reports on the above manuscript, and have reached a decision. The referees' comments are appended below, or you can access them online: please go to: ********

As you will see, the referees express considerable interest in your work, but have some significant criticisms and recommend a substantial revision of your manuscript before we can consider publication. If you are able to revise the manuscript along the lines suggested, which may involve further experiments, I will be happy receive a revised version of the manuscript. Your revised paper will be re-reviewed by one or more of the original referees, and acceptance of your manuscript will depend on your addressing satisfactorily the reviewers' major concerns. In particular there were a number of different analyses of what we expect is existing data that, if done, should provide more support for your model, or indicate that there is an alternative, and these should be performed. There are a few suggestions that involve new transgenic lines needed to support some claims. If you have made those lines already in the course of your work, inclusion is recommended, however, it may also be possible to soften the claims. Please feel free to write with questions about specific experiments and alternative in this regard.

Please also note that Development will normally permit only one round of major revision. If it would be helpful, you are welcome to contact us to discuss your revision in greater detail. Please send us a point-by-point response indicating your plans for addressing the referees’ comments, and we will look over this and provide further guidance.

© 2026. Published by The Company of Biologists under the terms of the Creative Commons Attribution License (https://creativecommons.org/licenses/by/4.0/).

Please attend to all of the reviewers' comments and ensure that you clearly highlight all changes made in the revised manuscript. Please avoid using 'Tracked changes' in Word files as these are lost in PDF conversion. I should be grateful if you would also provide a point-by-point response detailing how you have dealt with the points raised by the reviewers in the 'Response to Reviewers' box. If you do not agree with any of their criticisms or suggestions please explain clearly why this is so.

Reviewer 1

Advance summary and potential significance to field

The paper by Fitzsimons et al describes a very nicely crafted series of experiments addressing a fundamental question in plant developmental biology: how is the internal structure of the leaf formed to deliver distinct regions of cells characterised by different patterns of cells and air space? The topic falls clearly within the remit of "Development".

The authors use a well-described approach, combining quantitative analysis of cell and airspace development in Arabidopis (with a range of appropriate mutants) to first characterise the process of airspace formation, then test the hypothesis that growth or activity in the epidermis informs the process of airspace formation in the underlying mesophyll. There have been recent detailed descriptions of the process of mesophyll and airspace formation from the Ambrose group (Zhang et all, 2021) but these focused more on cell shape changes and did not address the issue of how the epidermis might inform mesophyll development, so the data reported here are distinct, advance the field and will be of interest to the readership of Development.

Comments for the author

I have a number of points that the authors should consider:

1) Although in very young leaves the designation of palisade and spongy mesophyll is relatively trivial (Fig 1A), in older leaves I would expect this to be not quite as simple. Can the authors say how they made the designation palisade vs spongy for calculation of porosity? The Methods section is not clear on this, indeed is generally very sparse on detail. Indeed, the method for cryoSEM (Fig 1A) is absent. I realise this image is essentially just for illustration, nevertheless it should be possible for a reader to reproduce all data shown, so I strongly recommend a more detailed Methods section (unless there is a supplementary section I have missed?). I think this is particularly important in the description of how cell areas and porosity were measured. There are appropriate references to the Kawase papers, but since this methodology is core to the paper, a brief summary at least would be very useful since, e.g., the confocal parameters used, objectives, etc, will have a significant outcome on data, plus there is almost always some level of user-selection in data selection/processing.

2) A major hypothesis in the paper is that the lateral (in-plane) growth of the epidermis essentially pulls the subtending mesophyll cells apart to influence the degree of airspace. Loss of brassinolide signalling leads to a surprising (novel) dip in palisade porosity later in leaf development (Fig. 4A). One (very reasonable) interpretation is that loss of brassinolide signalling decreases this lateral epidermal growth, leading to decreased palisade mesophyll airspace. An alternative hypothesis (explored line 176 onwards) is that in the brassinolide signalling mutant (bri1) there is an increased rate of cell division, leading to more mesophyll cells per volume, thus less airspace per volume. The data provided do not support this alternative hypothesis (as indicated by the authors).
Is there not a third alternative, that the loss of brassinolide signalling somehow influences the size of the mesophyll cells? In this scenario rate of division is unchanged and number of total cells is unchanged, but if those cells are all a bit larger, then you get less airspace per leaf volume? Eyeballing the data in Fig 4, I don't think this is the case (and is difficult to reconcile with a smaller leaf) but it would be nice to see the data (which I think the authors will have- they have calculated the number of palisade cells per leaf, so- with porosity data- should be able to estimate palisade cell size).

3) Along the same lines, when looking at the BigBrother data in Fig. 7, I think there is a change in palisade mesophyll cell size (cross-sectional area) after induction of BB (Fig 7F, top row). Is this

© 2026. Published by The Company of Biologists under the terms of the Creative Commons Attribution License (https://creativecommons.org/licenses/by/4.0/).

correct? As with point (2), graphing palisade cell size (cross-sectional area) would be informative. Is any change in cell cross-sectional area compensated (or exacerbated) by a change in z-depth (see point 4 below)?

4) The data presented in the paper focus on measurements of palisade/spongy cell cross-sectional area. From their confocal data, could the authors segment cells to get estimates of cell length (z-depth) and, thus, cell volume? Or estimates of total leaf depth? The data show very nicely that there are changes in cross-sectional cell area and total porosity, but it would be interesting to see if there was any compensation in growth in z, either at a cell level or in the whole leaf. Are we looking at changes in growth or changes in shape (i.e., how growth is distributed)? I suspect it is a combination, but any data the authors have to address this question would strengthen the paper.

5) Following on from (2) and (3), it's interesting that the Br mutants seem to show limited outcomes on the spongy mesophyll (Fig 3) whereas the BB mutant does influence the spongy layer (Fig 7), despite the activity (or loss) only being required in the epidermal layers to induce changes in the subtending mesophyll. Does this suggest that they might be utilising different pathways to influence mesophyll cell growth/division? I don't have an answer, but might be useful to raise in the Discussion.

Minor point
In Fig 5A the spongy data (yellow) are presented before the palisade (blue). Suggest reversing order on the graph so that it fits with rest of figure (palisade first, then spongy).

<u>Reviewer 2</u>

Advance summary and potential significance to field

One of the previous hypotheses regarding the patterning of leaf air spaces suggests that faster epidermal growth relative to the mesophyll pulls mesophyll cells apart, thereby expanding air spaces. Additionally, earlier studies, including work by the authors, have shown that BR signaling regulates (driving and/or inhibiting) growth from the epidermis, "instructing" the sub-epidermal layer. Building on this prior knowledge, the manuscript by Fitzsimons et al. proposes that BR signaling in the epidermis promotes the maintenance and/or expansion of air spaces in the Arabidopsis leaf. This is distinct from the initial formation of air spaces, which the authors show is unaffected by BR signaling. The message of the paper adds new knowledge.

Comments for the author

The manuscript presents a careful characterization of the leaf in various genetic backgrounds. However, some uncertainty remains regarding the conclusions made. I have several comments and suggestions that may help the authors strengthen their work:
1. The authors propose that faster epidermal growth relative to mesophyll cells promotes air space expansion. As evidence, they quantify air spaces in bri1 and report a temporal and cell-specific (palisade cells) reduction in air space expansion. They also show that epidermal BRI1 expression suppresses this phenotype. While these findings are compelling, what seems to be missing is a direct measurement of growth rates in the epidermis and mesophyll in these lines. For example, see: https://doi.org/10.1371/journal.pbio.2005952. Such measurements would directly test the core assumption of the paper. The epidermal expression of BB supports the idea that reduced epidermal growth can limit air space expansion, but this does not necessarily confirm that the reduced air space observed in the BR mutant are a result of slower epidermal growth relative to inner tissues.
2. Figure 6 - Regarding the number of cells in the leaf, previous studies have shown a reduction in both mesophyll and epidermal cell numbers in bri1 and BR biosynthesis mutants (e.g., https://doi.org/10.1111/nph.12036, https://doi.org/10.1111/nph.12036, note that a reduction in air spaces is also reported). In Figure 6D, the median cell number appears lower in the mutant, yet this difference is not statistically significant. In this regard, the manuscript does not specify the number of independent plants used across the experiments, which is essential for evaluating the robustness of the conclusions.

© 2026. Published by The Company of Biologists under the terms of the Creative Commons Attribution License (https://creativecommons.org/licenses/by/4.0/).

3. The slow growth observed in BR mutants is generally attributed to a slower cell cycle, so it is somewhat surprising that the authors report no effect on the rate of cell proliferation. The use of inducible cell marking is a nice approach. Perhaps it may have been more informative to perform this analysis at an earlier developmental stage than 8 days. Additionally, as with other experiments, the number of biological replicates is not stated, making it difficult to assess the robustness of the findings. Regarding the methodology, since the marked cells contain cytoplasmic GFP (I assume), how can the authors be certain that the GFP signal does not move between cells? Moreover, is there a control to ensure that neighboring GFP-expressing cells are not the result of independent cre/lox activation events rather than clonal expansion?

Additional comments:

4. Line 129: I am not familiar with bortezomib as a drug that inhibits BR biosynthesis. Did the authors mean to say brassinazole? The corresponding abbreviation is BRZ, and not BZR.
5. Lines 80-81, line 125: Perhaps I've missed it, but I did not find in Avery, 1933 a mentioning that the epidermis drives intercellular air space expansion.
6. Line 100. Nile red is first shown in Fig. 1 , need to add this Fig. as a reference too.
7. Line 237. Regarding the cell-autonomous effects of BR at the molecular level: please note that the study by Blanco-Touriñán et al. (2024) argues that BRI1 is not sufficient to drive growth from the epidermis alone, because the BRI1 open reading frame causes low-level expression in other cell types, which is necessary for full growth. Vragovic et al (https://doi.org/10.1073/pnas.1417947112) demonstrates that, while epidermal BRI1 influences growth in a non-cell-autonomous manner, gene expression remains largely autonomous.
8. It was previously shown by the authors that the BR mutant in U. gibba has reduced air spaces (this should be mentioned in the introduction). Does this imply that the epidermis grows faster than the inner cell layers in U. gibba stem as well?

<u>Reviewer 3</u>

Advance summary and potential significance to field

This interesting paper revisits the hypothesis of Avery (1933) that the epidermis in leaves drives the expansion of underlying mesophyll tissues. The Nile Red approach is nicely used to track intercellular air spaces alongside cell outlines over development. With a few additions (mostly imaging/quantification the authors likely already have), I think the manuscript will be even stronger and the palisade-versus-spongy narrative will land more cleanly.

Summary: The authors quantify leaf porosity over time and across BR perturbations: drug inhibition (BRZ), biosynthesis mutant (*dwf4*), and perception mutant (*bri1-116*). All reduce leaf growth and mesophyll porosity. Epidermis-specific rescue (*ATML1::BRI1*) *in bri1-116* restores porosity and leaf size, while epidermis-specific expression of the inhibitory protein BIG BROTHER (BB) in wild-type leaves reduces both. The authors conclude that epidermal growth non–cell-autonomously permits mesophyll air-space expansion, with the most obvious effects in the palisade layer. They argue this is likely mechanical rather than a signaling relay.

The paper is very interesting, clearly written, and well organized with clear figures. I have some suggestions to help strengthen the paper.

1. Cell size and porosity – Mesophyll structure is measured using porosity (%), which is not defined here, but I am assuming it is the percentage of tissue occupied by intercellular spaces, and that measurements are taken on area (2D images) and not volume (3D). Please clarify. The biggest thing that jumped out to me is that in addition to the effects on the intercellular spaces, the size of cells also show strong changes in all treatments, and this is clearly seen in both palisade and spongy layers. For example, looking at all the BR-related treatments in figures 3, 5, and 7, the palisade cells *and* spongy cells are both drastically reduced in area.

Relating to this, a major conclusion is that the palisade layer is the primary layer being affected by the treatments. Because the spongy cells are so branched, measurements of the area occupied by cell vs space in a given image are super tricky, and depend a lot on the plane of sectioning. In

© 2026. Published by The Company of Biologists under the terms of the Creative Commons Attribution License (https://creativecommons.org/licenses/by/4.0/).

the palisade cells, in most cases the images show the cells in a nice single layer, while the spongy cells are sectioned at varied depths along any given cell. Maybe this is why you went with the porosity measurements? The spongy cells seem to be smaller in the BR and BB experiments, possibly even proportionate to the reduction in the palisade cells. Air space size depends on cell expansion and size, so some quantification of cell size should be included in addition to the porosity, which, as a ratio, misses these details. If both layers are affected similarly, this would still support (actually strengthen) the overall conclusion that the epidermis controls the mesophyll.

2. Please show the epidermis - Since the proposed mechanism is epidermis-driven, it would be great to see the epidermis under each key condition. Do reductions in the epidermis match the reductions in the mesophyll? Are the reductions in epidermal growth the same on both sides (adaxial/abaxial) of the leaf? This doesn't need a new experimental approach necessarily, likely just re-slicing and quantifying from your existing stacks.

- For WT, *bri1-116, dwf4*, BRZ, *ATML1*::BRI1, and *ATML1*::BB, please include adaxial and abaxial epidermis images, plus:
    - Pavement-cell area, density, perimeter, and lobing (circularity/solidity).
    - Stomatal density/index (guard cell size optional).
- If possible, add a simple leaf curvature/flatness metric (side views + a curvature number). A dorsiventral growth imbalance would neatly connect epidermal mechanics to potential differential palisade/spongy outcomes.
- On growth mode: your mesophyll clonal analysis suggests division rates are stable in *bri1-116*. Do you see epidermal changes arising from division, expansion, or both? A brief note or proxy (e.g., epidermal cell density vs. size) would close the loop.

3. Confirm epidermis-only expression and that it stays epidermal across time - Because the epidermis-specific expression experiments are the main supporting evidence, it is very important to show that the expression is truly restricted to the epidermis, with absolutely zero protein in the inner tissues. This can be easily addressed by a few cross- sectional images. While the ATML1 promoter is the standard one to use for epidermal- specific expression studies, there are some important issues here to consider. (1) is expression of this promoter equal on both sides of the leaf? This also relates to the possible selective effects on the palisade layer. (2) True epidermis specificity of the epidermally expressed proteins. Movement of epidermally-expressed proteins through plasmodesmata into the mesophyll tissues is common, and depends (in my experience) a lot on the size of the expressed proteins and where in the cell they reside. For instance, nuclear-localized proteins like ATML1 itself are nicely sequestered in the nucleus and indeed shows clear epidermis expression, but cytosolic and even membrane proteins can leak. Additionally, the image of the leaf in figure 5C is low magnification, and contains the chloroplast channel, so it's hard to see the cells and tissue layers distinctly to confirm epidermis-specific signal. It will be important to include and/or replace this image with a higher resolution image showing an orthogonal re-slice through the z-stack (or better yet, an optical cross section of the leaf if possible to rule out the possibility of z-depth signal attenuation) with the separate channels shown in addition to the merged image. Secondly, epidermis-specific expression should ideally be shown from the very earliest stages of growth throughout the full observed time range.

Related to this, supplemental figure 3 does show a higher magnification image + orthogonal section for the *ATML1::BB-3XVENUS*. A few questions here. Did you choose triple Venus tagging in order to prevent leakage of the BIG BROTHER (BB) protein into the mesophyll? Second, can you describe briefly what the BB protein is? I looked it up because I had not heard of it. As a ubiquitin e3 ligase, shouldn't this protein be found predominately in the cytoplasm? From the image, the signal looks like plasma membrane (or cell wall). And it is almost exclusively restricted to the outer periclinal wall of the cells. Is this real or just strong signal attenuation from the sample with increasing depth? There is some yellow signal in the upper part of the image that sections obliquely through the leaf. What are the yellow structures? Some of it looks to possibly be in the mesophyll cells or air spaces. For this, it is also important to show the individual channels, because the chloroplast channel occludes the Venus channel too much.

© 2026. Published by The Company of Biologists under the terms of the Creative Commons Attribution License (https://creativecommons.org/licenses/by/4.0/).

4. The complementary experiment (mesophyll-specific expression of BRI1 and BB) - Assuming full epidermal specificity of the rescue experiments, the complementary experiment would greatly strengthen the paper. Specifically, does mesophyll-specific expression of BRI1-GFP fail to restore normal leaf growth of the *bri1* mutant as would be predicted? If it does, what would this mean? Similarly, does mesophyll-specific expression of BB-3XVenus fail to block leaf growth as would be predicted? I assume this work has been considered and is maybe already underway, so if possible, some kind of mesophyll-specific manipulation would greatly strengthen the paper.

Minor Points:

-Please cite and discuss the below paper, which is probably the most relevant study. It would be a nice point for the intro and then later in discussion. *Marcotrigiano, M. (2010). A role for leaf epidermis in the control of leaf size and the rate and extent of mesophyll cell division. American Journal of Botany, 97(2), Article 2.* htt ps:// doi.o rg/10 .37 32 / aj b.09 0010 2

-Please provide some background on what is known about BR's role in mesophyll

-For the violin plots in figure 3, can you please include data point overlays, like in your other plots?

- Replicates: For each dataset, please report biological n (leaves/plants), technical n (ROIs/cells), and the number of independent experimental repeats.- Some statistical annotations are confusing (e.g., "*** = P > 0.001" appears to be a typo in multiple figure legends). I think you mean P < 0.001?

- A model diagram summarizing the proposed mechanical interactions between epidermis and mesophyll may be helpful.

-Please add more details on image analysis and quantification, statistics, graphing software, and figure construction software.

-Please give some details on the nile red quantification in addition to the 'as described previously' (lines 305-306), since nobody enjoys having to go paper hunting to find the original description of a method.

-The cre-lox system is very nice. The schematics of the constructs in figures 6 and 7 are really helpful, but can you provide more explanation as to how it works and why you specifically used it? I gather that the short heat shock time (1-2 minutes) used for the clonal analysis (figure 6) compared to the long heat shock time (12 minutes) for the BB-Venus expression is why different times were used.
-There are a few mistakes and inconsistencies with the naming for the cre-lox vector constructs (understandable given the complexity of the constructs). For the cre-lox schematic in figure 7, I think the promoter shown should the pATML1 (as in the methods and supp table). Fig 7 shows p35S instead of pATML1 currently. There are also some inconsistencies with the nomenclature, terminators, and construct numbers:

BB construct (CW0091):

Methods: HS18.2:CRE-U5-Cre----**pAtML1::loxP-CyPET-t35S-loxP-BB-3xVenus-tAct**

Fig 7: proHS18:CRE-tHSP----p35S::loxP-CyPET- tACT2-loxP-BB-3xVenus-tAct2

Supp Table: HS18.2:CRE-U5-Cre-tHSP----pAtML1::loxP-CyPET-t35S-loxP-BB-3xVenus-tAct

Sector construct (EC71692) + (EC71 924) or (EC71028):

Methods: pHSP18.2::CRE-tAtHS construct (EC71692) and p35S-loxPrev-CyPET-t35S- loxPrev-GFP-tAct (EC71924).

© 2026. Published by The Company of Biologists under the terms of the Creative Commons Attribution License (https://creativecommons.org/licenses/by/4.0/).

Fig 6: pHSP18.2::CRE-t AtHS construct (EC71692) and p35S-loxP rev-CyPET-t35S-loxP rev- GFP-tAct (EC71924).

Supp Table: HS-CRE (71692) and p35S-loxP rev-CyPET-t35S-loxP rev-GFP-tAct (EC71028)

First revision

Author response to reviewers' comments

Reviewer 1:

The paper by Fitzsimons et al describes a very nicely crafted series of experiments addressing a fundamental question in plant developmental biology: how is the internal structure of the leaf formed to deliver distinct regions of cells characterised by different patterns of cells and air space? The topic falls clearly within the remit of "Development".

The authors use a well-described approach, combining quantitative analysis of cell and airspace development in Arabidopis (with a range of appropriate mutants) to first characterise the process of airspace formation, then test the hypothesis that growth or activity in the epidermis informs the process of airspace formation in the underlying mesophyll. There have been recent detailed descriptions of the process of mesophyll and airspace formation from the Ambrose group (Zhang et all, 2021) but these focused more on cell shape changes and did not address the issue of how the epidermis might inform mesophyll development, so the data reported here are distinct, advance the field and will be of interest to the readership of Development.

We thank the reviewer for the interest in our paper.

SUGGESTIONS TO AUTHORS

I have a number of points that the authors should consider:

1) Although in very young leaves the designation of palisade and spongy mesophyll is relatively trivial (Fig 1A), in older leaves I would expect this to be not quite as simple. Can the authors say how they made the designation palisade vs spongy for calculation of porosity? The Methods section is not clear on this, indeed is generally very sparse on detail. Indeed, the method for cryoSEM (Fig 1A) is absent. I realise this image is essentially just for illustration, nevertheless it should be possible for a reader to reproduce all data shown, so I strongly recommend a more detailed Methods section (unless there is a supplementary section I have missed?). I think this is particularly important in the description of how cell areas and porosity were measured. There are appropriate references to the Kawase papers, but since this methodology is core to the paper, a brief summary at least would be very useful since, e.g., the confocal parameters used, objectives, etc, will have a significant outcome on data, plus there is almost always some level of user-selection in data selection/processing.

We have now included information on cryoSEM in the methods (line 392-401), and added more detail to the methods throughout, including a more detailed summary of the Nile Red staining protocol (line 402-419).

For quantification of mesophyll porosity and cell morphology throughout development, we assigned palisade and spongy identity based primarily on position: palisade tissue is the layer of mesophyll underlying the adaxial epidermis, and spongy tissue is the layer of mesophyll underlying the abaxial epidermis (in actual fact Arabidopsis has three layers of spongy mesophyll cells, but we image only the subepidermal layer as this is most accessible). Spongy and palisade cells begin similarly shaped, and become more distinct as the leaf matures (visible in fig 2), but even mesophyll cells with similar morphologies can be accurately assigned to palisade or spongy based on the positional criteria outlined above. We have now made this clear in the methods (line 411-418).

© 2026. Published by The Company of Biologists under the terms of the Creative Commons Attribution License (https://creativecommons.org/licenses/by/4.0/).

2) A major hypothesis in the paper is that the lateral (in-plane) growth of the epidermis essentially pulls the subtending mesophyll cells apart to influence the degree of airspace. Loss of brassinolide signalling leads to a surprising (novel) dip in palisade porosity later in leaf development (Fig. 4A). One (very reasonable) interpretation is that loss of brassinolide signalling decreases this lateral epidermal growth, leading to decreased palisade mesophyll airspace. An alternative hypothesis (explored line 176 onwards) is that in the brassinolide signalling mutant (bri1) there is an increased rate of cell division, leading to more mesophyll cells per volume, thus less airspace per volume. The data provided do not support this alternative hypothesis (as indicated by the authors).

Is there not a third alternative, *that the loss of brassinolide signalling somehow influences the size of the mesophyll cells?* In this scenario rate of division is unchanged and number of total cells is unchanged, but if those cells are all a bit larger, then you get less airspace per leaf volume? Eyeballing the data in Fig 4, I don't think this is the case (and is difficult to reconcile with a smaller leaf) but it would be nice to see the data (which I think the authors will have- they have calculated the number of palisade cells per leaf, so- with porosity data- should be able to estimate palisade cell size).

We thank the reviewer for suggesting this third hypothesis, that lack of BR signalling reduces air space by increasing mesophyll size. We have now quantified palisade cell size in the bri1 mutant. This shows that bri1 palisade cells are significantly smaller than wild type (new figure S4). Combined with an overall smaller leaf this data is more consistent with the epidermal growth hypothesis. We have reworded the corresponding results section to highlight the potential role of both cell division and cell expansion in driving mesophyll growth and reducing air spaces in bri1, and that our data support the primary importance of the epidermis (lines 233-239).

3) Along the same lines, when looking at the BigBrother data in Fig. 7, I think there is a change in palisade mesophyll cell size (cross-sectional area) after induction of BB (Fig 7F, top row). Is this correct? As with point (2), graphing palisade cell size (cross- sectional area) would be informative. Is any change in cell cross-sectional area compensated (or exacerbated) by a change in z-depth (see point 4 below)?

We have now quantified palisade cell size in *AtML1::BB* lines. As with *bri1-116*, this shows that palisade cell size is smaller than wild type (fig S4) (Line 261-263).

4) The data presented in the paper focus on measurements of palisade/spongy cell cross-sectional area. From their confocal data, could the authors segment cells to get estimates of cell length (z-depth) and, thus, cell volume? Or estimates of total leaf depth? The data show very nicely that there are changes in cross-sectional cell area and total porosity, but it would be interesting to see if there was any compensation in growth in z, either at a cell level or in the whole leaf. Are we looking at changes in growth or changes in shape (i.e., how growth is distributed)? I suspect it is a combination, but any data the authors have to address this question would strengthen the paper.

Due to the technical difficulty of light penetration through mesophyll cells with Nile Red staining in living tissue our images do not span the full depth of a mesophyll cell. Therefore it is possible that loss of BR signalling alters growth orientation in 3D rather than volumetric growth rate, as has previously been shown in roots (https://www.nature.com/articles/s41477-021-01014-9). However, as air spaces form and expand primarily in the plane of the leaf this difference would not markedly affect our conclusions. As our existing images do not address this question, we have raised this possibility in the discussion (lines 309-315).

5) Following on from (2) and (3), it's interesting that the Br mutants seem to show limited outcomes on the spongy mesophyll (Fig 3) whereas the BB mutant does influence the spongy layer (Fig 7), despite the activity (or loss) only being required in the epidermal layers to induce changes in the subtending mesophyll. Does this suggest that they might be utilising different pathways to influence mesophyll cell growth/division? I don't have an answer, but might be useful to raise in the Discussion.

© 2026. Published by The Company of Biologists under the terms of the Creative Commons Attribution License (https://creativecommons.org/licenses/by/4.0/).

We have now extended the discussion section entitled "*Different developmental mechanisms control air space patterning in the spongy and palisade*" to combine data from *bri1* and *AtML1::BB* lines, and to speculate on the molecular mechanisms driving spongy and palisade tissue patterning (lines 333-349).

Minor point

In Fig 5A the spongy data (yellow) are presented before the palisade (blue). Suggest reversing order on the graph so that it fits with rest of figure (palisade first, then spongy).

We have now switched the spongy and palisade in fig 5 A so it is consistent with the rest of the figure.

Reviewer 2:

One of the previous hypotheses regarding the patterning of leaf air spaces suggests that faster epidermal growth relative to the mesophyll pulls mesophyll cells apart, thereby expanding air spaces. Additionally, earlier studies, including work by the authors, have shown that BR signaling regulates (driving and/or inhibiting) growth from the epidermis, "instructing" the sub-epidermal layer. Building on this prior knowledge, the manuscript by Fitzsimons et al. proposes that BR signaling in the epidermis promotes the maintenance and/or expansion of air spaces in the Arabidopsis leaf. This is distinct from the initial formation of air spaces, which the authors show is unaffected by BR signaling. The message of the paper adds new knowledge.

SUGGESTIONS TO AUTHORS

The manuscript presents a careful characterization of the leaf in various genetic backgrounds. However, some uncertainty remains regarding the conclusions made. I have several comments and suggestions that may help the authors strengthen their work:

1. The authors propose that faster epidermal growth relative to mesophyll cells promotes air space expansion. As evidence, they quantify air spaces in bri1 and report a temporal and cell-specific (palisade cells) reduction in air space expansion. They also show that epidermal BRI1 expression suppresses this phenotype. While these findings are compelling, what seems to be missing is a direct measurement of growth rates in the epidermis and mesophyll in these lines. For example, see: https://doi.org/10.1371/journal.pbio.2005952. Such measurements would directly test the core assumption of the paper. The epidermal expression of BB supports the idea that reduced epidermal growth can limit air space expansion, but this does not necessarily confirm that the reduced air space observed in the BR mutant are a result of slower epidermal growth relative to inner tissues.

We agree that live imaging differential growth between the palisade and epidermis would add additional weight to our conclusions. Unfortunately, this is technically challenging and has not been possible in our hands for a number of reasons. Firstly, the palisade mesophyll forms on the adaxial side of the leaf, which is pressed against the meristem throughout the early stages of development. This makes imaging an intact and growing leaf challenging. For this reason, previous work live imaging leaf growth and mesophyll morphogenesis (including the paper the reviewer refers to) have focussed on the abaxial side and the spongy mesophyll. The presence of trichomes on the adaxial epidermis also makes imaging challenging. For these reasons we chose to use other methods to infer growth dynamics during air space morphogenesis.

Several of our findings support the hypothesis that, in *bri1*, epidermal growth is reduced more than palisade mesophyll growth.

1- Epidermal growth is lower in the bri1 mutant (because overall leaf size is smaller)

2- Palisade mesophyll division is unchanged in the *bri1* mutant (based on our sector analysis in fig. 6, now extended earlier in leaf development)

© 2026. Published by The Company of Biologists under the terms of the Creative Commons Attribution License (https://creativecommons.org/licenses/by/4.0/).

3- Palisade mesophyll cell size is reduced in the *bri1* mutant (new data included in figure S4).
4- By measuring the change in porosity (% air space) through development, we have essentially quantified the relative difference in growth rate between the mesophyll and the overlying epidermis. If air spaces expand the mesophyll must be growing slower than the epidermis, whereas if air spaces shrink the mesophyll must be growing more quickly. This shows that:

In **wild type**, observed palisade mesophyll growth is lower than epidermal growth because intercellular air spaces expand throughout development.

In *bri1*, after air space initiation, palisade growth is higher than epidermal growth, as intercellular air spaces get smaller throughout development.

Together, the above data strongly support the hypothesis that reduced air space in bri1 is a result of slower epidermal growth relative to inner tissues. To make the strength of our evidence clear we have outlined the above reasoning more clearly in the results (lines 175-178 and 185-186).

2. Figure 6 - Regarding the number of cells in the leaf, previous studies have shown a reduction in both mesophyll and epidermal cell numbers in bri1 and BR biosynthesis mutants (e.g., https://doi.org/10.1111/nph.12036, https://doi.org/10.1111/nph.12036, note that a reduction in air spaces is also reported). In Figure 6D, the median cell number appears lower in the mutant, yet this difference is not statistically significant. In this regard, the manuscript does not specify the number of independent plants used across the experiments, which is essential for evaluating the robustness of the conclusions.

We have now included the number of independent plants used across experiments in all figures. In this case, at least 10 plants were used to count mesophyll cell number. Our results show the same trend as Zhiponova et al. but our leaves are smaller, and although we also find a slight reduction in cell number, it is not significant. These differences are likely due to differing growth conditions. We grow plants for these experiments on agar plates, whereas Zhiponova et al. grew them on peat pellets and obtained larger plants. It is possible that these larger plants allow them to identify differences that are less obvious at smaller leaf sizes. We have now cited Zhiponova et al., which also supports the reduction in mesophyll cell size in *bri1* that we describe (fig. S4, lines 236-237).

3. The slow growth observed in BR mutants is generally attributed to a slower cell cycle, so it is somewhat surprising that the authors report no effect on the rate of cell proliferation. The use of inducible cell marking is a nice approach. Perhaps it may have been more informative to perform this analysis at an earlier developmental stage than 8 days.

We have now performed sector analysis to quantify cell division rate at 4-7 days. Consistent with later time points, we observe no differences in palisade cell division at this point in leaf development (fig 6).

4. Additionally, as with other experiments, the number of biological replicates is not stated, making it difficult to assess the robustness of the findings. Regarding the methodology, since the marked cells contain cytoplasmic GFP (I assume), how can the authors be certain that the GFP signal does not move between cells? Moreover, is there a control to ensure that neighboring GFP-expressing cells are not the result of independent cre/lox activation events rather than clonal expansion?

Previous work from ourselves and others has shown that the cytoplasmic GFP used in indudible CRE/lox sector lines is cell autonomous and can be used to accurately quantify sector size and cell number (eg. Lee et al. 2018 https://doi.org/10.1371/journal.pbio.3000427, Kuchen et al. 2012 10.1126/science.1214678, Sauret-Gueto et al. 2013 https://doi.org/10.1371/journal.pbio.1001550). If GFP were mobile we would expect a gradual diluting of GFP signal in the cells surrounding the sector, and we have never observed this (as can be observed in fig. 6 and in previous work from ourselves and others (eg. 10.1371/journal.pbio.3000427, https://doi.org/10.1126/science.1214678)

There is no control to ensure that neighbouring cells within a clone are not the result of

© 2026. Published by The Company of Biologists under the terms of the Creative Commons Attribution License (https://creativecommons.org/licenses/by/4.0/).

independent activation events rather than clonal expansion. However, we induce sectors at a low level (4 or 5 per leaf), and this makes induction of neighbouring cells unlikely. Furthermore, induction of two neighbouring cells would give a sector with twice as many cells compared to a sector that arises from a single cell. This would give our data a bimodal distribution. At the density of sectors used in these experiments we do not observe this, so we are reasonably confident that our sectors are fully clonal and reflect cell division of a single cell.

Additional comments:

4. Line 129: I am not familiar with bortezomib as a drug that inhibits BR biosynthesis. Did the authors mean to say brassinazole? The corresponding abbreviation is BRZ, and not BZR.

Thank you for noticing this mistake. We have corrected the abbreviation throughout.

5. Lines 80-81, line 125: Perhaps I've missed it, but I did not find in Avery, 1933 a mentioning that the epidermis drives intercellular air space expansion.

Avery (1933) states that the epidermal tissue pulls apart mesophyll cells in both layers to drive intercellular air space morphogenesis:

> *"cells of the epidermis continue enlarging after those of the middle and lower mesophyll have ceased to enlarge. As a consequence (having ceased division only a very short time after the epidermal cells), they are pulled apart as the epidermal cells continue to enlarge. This results in the spongy tissue… the palisade cells (which are the last to cease dividing) are pulled apart in a similar fashion by the enlarging upper epidermal cells, though not as markedly as in the case of the spongy tissues." (Page 571, paragraph 3 and 4)*

6. Line 100. Nile red is first shown in Fig. 1 , need to add this Fig. as a reference too.

We have now included fig 1 as a reference here.

7. Line 237. Regarding the cell-autonomous effects of BR at the molecular level: please note that the study by Blanco-Touriñán et al. (2024) argues that BRI1 is not sufficient to drive growth from the epidermis alone, because the BRI1 open reading frame causes low-level expression in other cell types, which is necessary for full growth. Vragovic et al (https://doi.org/10.1073/pnas.1417947112) demonstrates that, while epidermal BRI1 influences growth in a non-cell-autonomous manner, gene expression remains largely autonomous.

We agree with the reviewer that it is possible that our epidermal rescue results are due to a low level of expression in the mesophyll driven by the BRI1 open reading frame, as described in Blanco-Touriñán *et al.* (2024). We have now highlighted this possibility clearly (lines 198-202). In addition, our results are consistent with both Blanco- Touriñán *et al.* (2024) and Vragovic *et al.* 2015: we also propose that the molecular action of BRI1 is predominantly cell autonomous, while the effects on mesophyll patterning are due to the non-cell autonomous effects of differential growth. We have now discussed this distinction and the commonalities with previous work in the discussion (lines 293-304).

To test whether BRI1 can act cell autonomously to rescue cell size and air space patterning in the palisade mesophyll we also now include new experiments where we induce clonal rescue sectors of BRI1-VENUS in the *bri1-116* mutant. When induced constitutively this construct rescues leaf size and mesophyll patterning (fig 5G and H), but when expressed in sectors it does not confer cell-autonomous rescue of mesophyll cell size, and mesophyll cells remain tightly packed (fig 5G, H and I). This is further evidence that BRI1 acts to regulate air space patterning indirectly by promoting growth in the epidermis. These data are included in a new results section (lines 196-213) and discussed in the discussion (lines 293-304). Interestingly, these data also show that BRI1 is not expressed at all unless it is adjacent to a promoter (as uninduced sector lines do not show BRI1-VENUS expression or morphological rescue). Therefore, it is likely that the open reading frame regulatory element(s) act as an enhancer, rather than an autonomous low level promoter.

8. It was previously shown by the authors that the BR mutant in U. gibba has reduced air spaces (this should be mentioned in the introduction). Does this imply that the epidermis grows faster

© 2026. Published by The Company of Biologists under the terms of the Creative Commons Attribution License (https://creativecommons.org/licenses/by/4.0/).

than the inner cell layers in U. gibba stem as well?

As we highlighted in the introduction, we previously showed that in the *dwf4* mutant of *U. gibba* the internal cell layers grow faster than the epidermis (Kelly-Bellow et al. 2023). In wild type *U. gibba* it is possible that the epidermis grows either equal to, or faster than the internal tissue. Our computational models in that paper begin after air space formation and assume equal growth between the two layers.

We have now included the fact that the *dwf4* mutant in U. gibba has reduced stem air spaces as part of our project rationale in the introduction:

As the epidermis has been proposed to drive intercellular air space expansion (Avery, 1933), and the BR synthesis mutant Ugdwf4 in U. gibba has reduced intercellular air spaces in stems (Kelly-Bellow et al., 2023), we hypothesised that BR promotes epidermal growth in leaves to drive air space formation and patterning. (lines 90-93)

Reviewer 3:

This interesting paper revisits the hypothesis of Avery (1933) that the epidermis in leaves drives the expansion of underlying mesophyll tissues. The Nile Red approach is nicely used to track intercellular air spaces alongside cell outlines over development. With a few additions (mostly imaging/quantification the authors likely already have), I think the manuscript will be even stronger and the palisade-versus-spongy narrative will land more cleanly.

Summary: The authors quantify leaf porosity over time and across BR perturbations: drug inhibition (BRZ), biosynthesis mutant (dwf4), and perception mutant (bri1-116). All reduce leaf growth and mesophyll porosity. Epidermis-specific rescue (ATML1::BRI1) in bri1-116 restores porosity and leaf size, while epidermis-specific expression of the inhibitory protein BIG BROTHER (BB) in wild-type leaves reduces both. The authors conclude that epidermal growth non-cell-autonomously permits mesophyll air-space expansion, with the most obvious effects in the palisade layer. They argue this is likely mechanical rather than a signaling relay.

The paper is very interesting, clearly written, and well organized with clear figures. I have some suggestions to help strengthen the paper.

We thank the reviewer for their kind comments.

1. Cell size and porosity – Mesophyll structure is measured using porosity (%), which is not defined here, but I am assuming it is the percentage of tissue occupied by intercellular spaces, and that measurements are taken on area (2D images) and not volume (3D). Please clarify.

We define porosity as percentage airspace measured in 2D, and have now included this description in both the results (line 108) and methods (line 411-412).

2. The biggest thing that jumped out to me is that in addition to the effects on the intercellular spaces, the size of cells also show strong changes in all treatments, and this is clearly seen in both palisade and spongy layers. For example, looking at all the BR-related treatments in figures 3, 5, and 7, the palisade cells and spongy cells are both drastically reduced in area.

   Relating to this, a major conclusion is that the palisade layer is the primary layer being affected by the treatments. Because the spongy cells are so branched, measurements of the area occupied by cell vs space in a given image are super tricky, and depend a lot on the plane of sectioning. In the palisade cells, in most cases the images show the cells in a nice single layer, while the spongy cells are sectioned at varied depths along any given cell. Maybe this is why you went with the porosity measurements? The spongy cells seem to be smaller in the BR and BB experiments, possibly even proportionate to the reduction in the palisade cells. Air space size depends on cell expansion and size, so some quantification of cell size should be included in addition to the porosity, which, as

© 2026. Published by The Company of Biologists under the terms of the Creative Commons Attribution License (https://creativecommons.org/licenses/by/4.0/).

a ratio, misses these details. If both layers are affected similarly, this would still support (actually strengthen) the overall conclusion that the epidermis controls the mesophyll.

We quantify porosity rather than cell morphology as in this paper we are primarily interested in air space morphogenesis rather than cell morphogenesis. To address the reviewer's point, we have now quantified palisade and epidermal cell size in WT, *bri1-116* and *AtML1::BB* (fig S4, lines 233-239, 261-263). These data show that cells in both layers are smaller. Unfortunately, due to the highly irregular 3D shapes of spongy mesophyll cells, we were unable to confidently quantify spongy mesophyll size in our 2D images, so have not included this data.

2. Please show the epidermis - Since the proposed mechanism is epidermis-driven, it would be great to see the epidermis under each key condition. Do reductions in the epidermis match the reductions in the mesophyll? Are the reductions in epidermal growth the same on both sides (adaxial/abaxial) of the leaf? This doesn't need a new experimental approach necessarily, likely just re-slicing and quantifying from your existing stacks.

• For WT, bri1-116, dwf4, BRZ, ATML1::BRI1, and ATML1::BB, please include adaxial and abaxial epidermis images, plus:

o Pavement-cell area, density, perimeter, and lobing (circularity/solidity).
o Stomatal density/index (guard cell size optional).

Our proposed mechanism requires reduced growth in the epidermis at the tissue level, and could be driven by cell expansion, cell division, or a combination of both. The important factor is a reduction in overall epidermal size, which we see in the reduced size of leaves in both *bri1-116* and *AtML1::BB*. We now highlight this point more clearly (line 185-186)

To better understand the cellular basis of differential growth, we have now quantified cell size in the adaxial epidermis of 21 day old leaves of WT, bri1-116 and AtML1::BB (fig. S4B and C), as well as stomatal density (fig. S4A). Alterations in cell size in the epidermis are consistent with those previously described in *bri1* (https://nph.onlinelibrary.wiley.com/doi/10.1111/nph.12036), and confirm the increase in stomatal density in BR mutants, as has previously been described (eg. https://pmc.ncbi.nlm.nih.gov/articles/PMC3292258/ https://www.frontiersin.org/journals/plant-science/articles/10.3389/fpls.2024.1377352/full). We now refer to these results in our results (line 233-239, 261-263) and discussion, integrating the role of stomata signalling and differential growth (line 354-363). We have not quantified the abaxial epidermis as our existing data did not contain the epidermal layer and the paper focuses on the adaxial mesophyll.

We have also now highlighted the hypothesis that differential growth between the upper and lower epidermises underlies air space patterning in the palisade and spongy (lines 344-349). (see answer to the below point)

• If possible, add a simple leaf curvature/flatness metric (side views + a curvature number). A dorsiventral growth imbalance would neatly connect epidermal mechanics to potential differential palisade/spongy outcomes.

We agree that a dorsiventral growth imbalance would neatly connect epidermal mechanics to differential effects on the spongy and epidermis. As the reviewer implies, a change in curvature from flat (as in wild type) to upward curving would suggest that growth is preferentially reduced in the upper epidermis (as in Coen and Rebocho, 2016 https://doi.org/10.1016/j.devcel.2016.09.006). Leaves of the *bri1-116* mutant do not curve upwards (visible in fig. S2), suggesting that lower growth on the upper epidermis is unlikely. We have now discussed the hypothesis that differential growth between upper and lower epidermis accounts for differential effects on spongy and palisade air space patterning (line 344-349).

• On growth mode: your mesophyll clonal analysis suggests division rates are stable in bri1-116. Do you see epidermal changes arising from division, expansion, or both? A brief note or proxy

© 2026. Published by The Company of Biologists under the terms of the Creative Commons Attribution License (https://creativecommons.org/licenses/by/4.0/).

(e.g., epidermal cell density vs. size) would close the loop.

Previous work has shown that the epidermal cells of *bri1* are smaller and reduced in number (eg. Zhiponova et al. 2012 https://doi.org/10.1111/nph.12036). This suggests that both cell expansion and division are reduced in the epidermis, with the greatest effect on cell expansion. We have also now quantified epidermal cell size (see response to point 2 above) and show that, consistent with published work, epidermal cell size is reduced (fig S4, line 233-239)

3. Confirm epidermis-only expression and that it stays epidermal across time -

Because the epidermis-specific expression experiments are the main supporting evidence, it is very important to show that the expression is truly restricted to the epidermis, with absolutely zero protein in the inner tissues. This can be easily addressed by a few cross-sectional images. While the ATML1 promoter is the standard one to use for epidermal-specific expression studies, there are some important issues here to consider.

> 3-1-is expression of this promoter equal on both sides of the leaf? This also relates to the possible selective effects on the palisade layer.

The expression of *AtML1* is well described as highly specific to the epidermis throughout the entire plant, including both adaxial and abaxial leaf surfaces to a similar level (eg. Sessions et al. 2002 (https://doi.org/10.1046/j.1365-313x.1999.00594.x) and Lu et al. 1996 (https://doi.org/10.1105/tpc.8.12.2155)).

As our work is focussed on the palisade, we show images of the adaxial epidermis and palisade mesophyll and confirm YFP localisation in the epidermis (fig S3). We have included a new image with refined imaging parameters to remove any reflectance, and believe that this now clearly shows epidermal specificity.

> 3-2-True epidermis specificity of the epidermally expressed proteins. Movement of epidermally-expressed proteins through plasmodesmata into the mesophyll tissues is common, and depends (in my experience) a lot on the size of the expressed proteins and where in the cell they reside. For instance, nuclear- localized proteins like ATML1 itself are nicely sequestered in the nucleus and indeed shows clear epidermis expression, but cytosolic and even membrane proteins can leak.

Using the AtML1 promoter to specifically induce expression in the epidermis is a well- established approach that has been used previously by other labs. For example, Bemis et al. (https://doi.org/10.1016/j.ydbio.2006.12.049) used the ATML1 promoter to drive cell layer-specific changes in cell division. Our approach is in line with theirs and, to further limit expression of BB to the epidermis we tagged it with three fluorescent proteins (as the reviewer suggests in 3-4 below). A triple fluorescent protein tag has previously been shown to restrict intercellular movement between cell layers in the leaf (https://doi.org/10.1016/j.cub.2013.03.044, see answer to point 3-3 below). We have also confirmed epidermal-specific Venus expression via confocal imaging (fig S3). Although very low levels of expression are impossible to rule out, we have a sufficient level of evidence to support epidermal-specific expression, and more evidence than provided in previously published work of this type.

> 3-3-Additionally, the image of the leaf in figure 5C is low magnification, and contains the chloroplast channel, so it's hard to see the cells and tissue layers distinctly to confirm epidermis-specific signal. It will be important to include and/or replace this image with a higher resolution image showing an orthogonal re-slice through the z-stack (or better yet, an optical cross section of the leaf if possible to rule out the possibility of z-depth signal attenuation) with the separate channels shown in addition to the merged image.

We have now provided a clearer image with separate chloroplast and YFP channels confirming epidermal expression of AtML1::BB (fig S3)

© 2026. Published by The Company of Biologists under the terms of the Creative Commons Attribution License (https://creativecommons.org/licenses/by/4.0/).

3-4 -Secondly, epidermis-specific expression should ideally be shown from the very earliest stages of growth throughout the full observed time range. Related to this, supplemental figure 3 does show a higher magnification image + orthogonal section for the ATML1::BB-3XVENUS. A few questions here. Did you choose triple Venus tagging in order to prevent leakage of the BIG BROTHER (BB) protein into the mesophyll?

As the reviewer suggests, we chose to tag BB with the 3xVenus as tagging with triple fluorescent proteins has previously been used successfully to restrict intercellular movement of a mobile protein (AN3) between the epidermis and the mesophyll (https://doi.org/10.1016/j.cub.2013.03.044). We have now made this rationale clear in the methods (line 438-440) and results (line 253-255).

As the AtML1 promoter is well-described to be epidermal specific throughout development (see response to point 3-3 above) we have not included images of epidermal-specific expression at early developmental timepoints. However, we believe that our new image combined with previous work showing that AtML1 expression is specific to the epidermis and sustained throughout development makes it overwhelmingly likely that AtML1::BB is restricted to the epidermis.

3-5- Second, can you describe briefly what the BB protein is? I looked it up because I had not heard of it. As a ubiquitin e3 ligase, shouldn't this protein be found predominately in the cytoplasm? From the image, the signal looks like plasma membrane (or cell wall). And it is almost exclusively restricted to the outer periclinal wall of the cells. Is this real or just strong signal attenuation from the sample with increasing depth? There is some yellow signal in the upper part of the image that sections obliquely through the leaf. What are the yellow structures? Some of it looks to possibly be in the mesophyll cells or air spaces. For this, it is also important to show the individual channels, because the chloroplast channel occludes the Venus channel too much.

We have now given more detail on the described role of BB (lines 251-253) and provided new images of leaves expressing AtML1::BB to further confirm epidermal expression, including images of each fluorescent channel separately. We have refined our imaging approach to reduce any non-specific fluorescence or reflectance, and allow the localisation of BB to be better seen (fig. S3). The yellow structures adjacent to the mesophyll in our original image that the reviewer refers to were stomata either expressing YFP or autofluorescing. Our new images show the localisation of AtML1::BB much more clearly, and we thank the reviewer for encouraging us to do this.

4. The complementary experiment (mesophyll-specific expression of BRI1 and BB) - Assuming full epidermal specificity of the rescue experiments, the complementary experiment would greatly strengthen the paper. Specifically, does mesophyll-specific expression of BRI1-GFP fail to restore normal leaf growth of the bri1 mutant as would be predicted? If it does, what would this mean? Similarly, does mesophyll-specific expression of BB-3XVenus fail to block leaf growth as would be predicted? I assume this work has been considered and is maybe already underway, so if possible, some kind of mesophyll-specific manipulation would greatly strengthen the paper.

To test whether mesophyll-specific BRI1 can rescue palisade tissue patterning we have now included data showing heatshock-inducible clonal CRE/lox sectors of BRI1 in the mesophyll (fig. 5F-I). When BRI1 expression is induced constitutively at 3 DAS, it rescues overall leaf size and air space patterning in mature leaves (fig. G and H), but when it is induced in clonal sectors in the palisade at 3DAS, it does not confer cell autonomous rescue of air spaces or cell size despite active BRI1 signalling in these cells (fig 5G, H and I) (lines 198-215). This shows that BR signalling in the palisade is unable to drive cell expansion and air space patterning here, and further supports the hypothesis that BR signalling acts primarily in the epidermis to control air space patterning non-cell autonomously via differential growth rather than molecular signals.

Minor Points:

-Please cite and discuss the below paper, which is probably the most relevant study. It would be

© 2026. Published by The Company of Biologists under the terms of the Creative Commons Attribution License (https://creativecommons.org/licenses/by/4.0/).

a nice point for the intro and then later in discussion. Marcotrigiano, M. (2010). A role for leaf epidermis in the control of leaf size and the rate and extent of mesophyll cell division. American Journal of Botany, 97(2), Article 2.
https://doi.org/10.3732/ajb.0900102

Thanks for encouraging us to discuss this interesting paper! We have now included this paper in the introduction (lines 76-81) and discussion (line 291-292)

-Please provide some background on what is known about BR's role in mesophyll

Very little is known about the specific role of BR in the mesophyll. To our knowledge the closest example is in regulating chloroplast area in the bundle sheath (Cackett et al. 2025 - https://doi.org/10.1093/plphys/kiaf108). We have now included a brief discussion of this paper and potential cell-autonomous roles for BR in the mesophyll (lines 304-308).

-For the violin plots in figure 3, can you please include data point overlays, like in your other plots?

We have now included data point overlays in fig 3.

- Replicates: For each dataset, please report biological n (leaves/plants), technical n (ROIs/cells), and the number of independent experimental repeats.- Some statistical annotations are confusing (e.g., "*** = P > 0.001" appears to be a typo in multiple figure legends). I think you mean P < 0.001?

We have now reported biological and technical replicates as well as the numbers of experimental repeats, as well as clarifying the statistical annotations throughout.

- A model diagram summarizing the proposed mechanical interactions between epidermis and mesophyll may be helpful.

We have now included a model diagram to summarise interactions between epidermis and mesophyll in wild type and in our layer-specific manipulations (fig. 8).

-Please add more details on image analysis and quantification, statistics, graphing software, and figure construction software.

We have added additional details throughout for the methods including Nile Red quantification (lines 405-419), sector induction (lines 447-452), statistical methods and figure construction (lines 467-471) as well as details on SEM (line 392-401).

-Please give some details on the nile red quantification in addition to the 'as described previously' (lines 305-306), since nobody enjoys having to go paper hunting to find the original description of a method.

We have included more details on how to perform nile red staining, including the thresholding images to obtain porosity measurements (lines 409-412).

-The cre-lox system is very nice. The schematics of the constructs in figures 6 and 7 are really helpful, but can you provide more explanation as to how it works and why you specifically used it? I gather that the short heat shock time (1-2 minutes) used for the clonal analysis (figure 6) compared to the long heat shock time (12 minutes) for the BB- Venus expression is why different times were used.

We used CRE-lox recombination to induce transgenes because in our hands it is a reliable way to induce gene expression and it is a well-established way to test whether gene function acts cell autonomously in a range of species (eg. https://doi.org/10.3791/56675).

We used it to induce GFP and perform sector analysis as it is an effective method of estimating cell division rates without live imaging, which is technically challenging in internal tissues. We

© 2026. Published by The Company of Biologists under the terms of the Creative Commons Attribution License (https://creativecommons.org/licenses/by/4.0/).

have now included a rationale for long and short heat shock times in the methods (lines 447-452).

-There are a few mistakes and inconsistencies with the naming for the cre-lox vector constructs (understandable given the complexity of the constructs). For the cre-lox schematic in figure 7, I think the promoter shown should the pATML1 (as in the methods and supp table). Fig 7 shows p35S instead of pATML1 currently. There are also some inconsistencies with the nomenclature, terminators, and construct numbers:

BB construct (CW0091):

Methods: HS18.2:CRE-U5-Cre----pAtML1::loxP-CyPET-t35S-loxP-BB-3xVenus-tAct

Fig 7: proHS18:CRE-tHSP----p35S::loxP-CyPET-tACT2-loxP-BB-3xVenus-tAct2

Supp Table: HS18.2:CRE-U5-Cre-tHSP----pAtML1::loxP-CyPET-t35S-loxP-BB-3xVenus- tAct

Sector construct (EC71692) + (EC71924) or (EC71028):

Methods: pHSP18.2::CRE-tAtHS construct (EC71692) and p35S-loxPrev-CyPET- t35SloxPrev-GFP-tAct (EC71924).

Fig 6: pHSP18.2::CRE-tAtHS construct (EC71692) and p35S-loxPrev-CyPET-t35S- loxPrevGFP-tAct (EC71924).

Supp Table: HS-CRE (71692) and p35S-loxPrev-CyPET-t35S-loxPrev-GFP-tAct (EC71028)

We have now made the construct labelling consistent throughout, including correctly labelling the AtML1 promoter in figure 7 (thank you for noticing this!). We have also included new construct layouts for the new BRI1-VENUS sector line (lines 461-466,Table S1).

Second decision letter

MS ID#: dev.205110R1

MS TITLE: Brassinosteroid controls leaf air space patterning non-cell autonomously by promoting epidermal growth

AUTHORS: James M. Fitzsimons, Ana B. Rock, Richard L. De Falbe, Samantha Fox and Chris D. Whitewoods

Dear Dr Whitewoods,

I have now received all the referees reports on the above manuscript, and have reached a decision. The referees' comments are appended below.

The overall evaluation is positive and we would like to publish a revised manuscript in Development, provided that the referees' comments can be satisfactorily addressed. There are only a few text changes required, but I do think that these changes will improve the manuscript, and they will be quite straightforward to complete. If you do not agree with any of their criticisms or suggestions explain clearly why this is so in a response to reviewers.

Reviewer 1

Advance summary and potential significance to field

© 2026. Published by The Company of Biologists under the terms of the Creative Commons Attribution License (https://creativecommons.org/licenses/by/4.0/).

The paper by Fitzsimons et al describes a very nicely crafted series of experiments addressing a fundamental question in plant developmental biology: how is the internal structure of the leaf formed to deliver distinct regions of cells characterised by different patterns of cells and air space? The topic falls clearly within the remit of "Development".

The authors use a well-described approach, combining quantitative analysis of cell and airspace development in Arabidopis (with a range of appropriate mutants) to first characterise the process of airspace formation, then test the hypothesis that growth or activity in the epidermis informs the process of airspace formation in the underlying mesophyll. There have been recent detailed descriptions of the process of mesophyll and airspace formation from the Ambrose group (Zhang et all, 2021) but these focused more on cell shape changes and did not address the issue of how the epidermis might inform mesophyll development, so the data reported here are distinct, advance the field and will be of interest to the readership of Development.

Comments for the author

The authors have addressed all of the points I raised in the original review.

Reviewer 2

Advance summary and potential significance to field

The current version is much improved, and the authors present a very nice study.
I have only minor comments:
1) Regarding my previous comment # 7: "Please note that the study by Blanco-Touriñán et al. (2024) argues that BRI1 is not sufficient to drive growth from the epidermis alone…."
Here the authors show that no low-level expression of BRI1 is detected outside the tissue of interest, and that any trace expression of BRI1 in the mesophyll (if present) is not sufficient to drive growth, whereas epidermal BRI1 is. This is adequately presented.
Note that residual expression levels of BRI1 outside the epidermis needed for growth were the main result that led Blanco-Touriñán et al. to conclude that BRI1 regulation is autonomous, which is not in line with the conclusions here. Thus, in my opinion, the sentence in lines 291-292 should be omitted, and the paragraph could start directly with: "Our work here suggests that the non-cell…"
2) Vragović et al. is well cited in this context but is missing from the reference list.
3) Clicking on the high-resolution version of Figure 5 opens Figure 6, likely due to an uploading error.

Reviewer 4

Advance summary and potential significance to field

The manuscript by Fitzsimons et al., addresses a century-old question in plant developmental biology: how are intercellular air spaces patterned in leaves? The authors identified brassinosteroid (BR) signaling as the regulator of air space patterning in the palisade, and demonstrated that this regulation operates non-cell-autonomously through epidermal growth. The revision is substantially improved over the prior version. The response to reviewers is thorough and the authors have genuinely addressed most concerns with new data, including the clonal BRI1 sector analysis, rather than just reframing existing arguments. However, several issues remain to be addressed before final acceptance.

1. The authors added new clonal BRI1 sector data (Fig. 5F-I) to show that mesophyll clonal sectors express BRI1-VENUS (line 204) but that palisade cells remain tightly packed and cell size is not rescued (Fig. 5G, H, I), which is particularly compelling. However, looking at the methods (lines 454-456), clonal sectors were identified by the absence of RFP fluorescence because "BRI1-GFP fluorescence was dim and often only visible at 20x magnification with tau gating." This raises a concern: if the BRI1-VENUS signal is very dim, how confident are the authors that the sectors are actually expressing sufficient BRI1 to activate downstream BR signaling? The absence of a rescue could reflect insufficient BRI1 expression in sectors. It will be great if the authors could provide

© 2026. Published by The Company of Biologists under the terms of the Creative Commons Attribution License (https://creativecommons.org/licenses/by/4.0/).

evidence that BRI1-VENUS-expressing sectors show detectable activation of BR signaling (e.g. using a BR signaling reporter?), or at least discuss this limitation.

2. Figure 7 discrepancy: The text (line 253-254) states spongy porosity was reduced in AtML1::BB, but the title of Figure 7 says "reduces palisade, but not spongy mesophyll porosity." This contradiction needs resolving — the data in Fig. 7C shows statistically significant reduction in spongy porosity too, making both the figure title and the result potentially misleading.

3. Lines 257-258, the authors state that the AtML1::BB data show that "reducing epidermal growth can reduce air space expansion in the mesophyll tissue non-cell autonomously, independently of stomata." This conclusion is stated too confidently. It is true that AtML1::BB increases stomatal density (Fig. S4A), however the paper does not demonstrate that air space reduction in AtML1::BB is independent of stomata. Please soften the statement.

4, Line 433 refers to "CW0008" which appears to be an error for "CW0153" (the inducible BRI1 sector line). The entire Methods section should be checked carefully for similar errors.

Second revision

Author response to reviewers' comments

Response to reviewers 2:

Reviewer 1: SUMMARY OF THE ADVANCE MADE IN THIS PAPER AND ITS POTENTIAL SIGNIFICANCE TO THE FIELD

The paper by Fitzsimons et al describes a very nicely crafted series of experiments addressing a fundamental question in plant developmental biology: how is the internal structure of the leaf formed to deliver distinct regions of cells characterised by different patterns of cells and air space? The topic falls clearly within the remit of "Development".

The authors use a well-described approach, combining quantitative analysis of cell and airspace development in Arabidopis (with a range of appropriate mutants) to first characterise the process of airspace formation, then test the hypothesis that growth or activity in the epidermis informs the process of airspace formation in the underlying mesophyll. There have been recent detailed descriptions of the process of mesophyll and airspace formation from the Ambrose group (Zhang et all, 2021) but these focused more on cell shape changes and did not address the issue of how the epidermis might inform mesophyll development, so the data reported here are distinct, advance the field and will be of interest to the readership of Development.

SUGGESTIONS TO AUTHORS

The authors have addressed all of the points I raised in the original review.

Thank you!

Reviewer 2:

The current version is much improved, and the authors present a very nice study. I have only minor comments:

1) Regarding my previous comment # 7: "Please note that the study by Blanco-Touriñán et al. (2024) argues that BRI1 is not sufficient to drive growth from the epidermis alone…."

Here the authors show that no low-level expression of BRI1 is detected outside the tissue of interest, and that any trace expression of BRI1 in the mesophyll (if present) is not sufficient to

© 2026. Published by The Company of Biologists under the terms of the Creative Commons Attribution License (https://creativecommons.org/licenses/by/4.0/).

drive growth, whereas epidermal BRI1 is. This is adequately presented.

Note that residual expression levels of BRI1 outside the epidermis needed for growth were the main result that led Blanco-Touriñán et al. to conclude that BRI1 regulation is autonomous, which is not in line with the conclusions here. Thus, in my opinion, the sentence in lines 291-292 should be omitted, and the paragraph could start directly with: "Our work here suggests that the non-cell…"

We have now removed this line.

2) Vragović et al. is well cited in this context but is missing from the reference list.

Thank you for noticing this. We have now added Vragović et al. to the reference list.

3) Clicking on the high-resolution version of Figure 5 opens Figure 6, likely due to an uploading error.

We have now corrected the figure order.

Reviewer 4: The manuscript by Fitzsimons et al., addresses a century-old question in plant developmental biology: how are intercellular air spaces patterned in leaves? The authors identified brassinosteroid (BR) signaling as the regulator of air space patterning in the palisade, and demonstrated that this regulation operates non-cell-autonomously through epidermal growth. The revision is substantially improved over the prior version. The response to reviewers is thorough and the authors have genuinely addressed most concerns with new data, including the clonal BRI1 sector analysis, rather than just reframing existing arguments. However, several issues remain to be addressed before final acceptance.

1. The authors added new clonal BRI1 sector data (Fig. 5F-I) to show that mesophyll clonal sectors express BRI1-VENUS (line 204) but that palisade cells remain tightly packed and cell size is not rescued (Fig. 5G, H, I), which is particularly compelling. However, looking at the methods (lines 454-456), clonal sectors were identified by the absence of RFP fluorescence because "BRI1-GFP fluorescence was dim and often only visible at 20x magnification with tau gating." This raises a concern: if the BRI1-VENUS signal is very dim, how confident are the authors that the sectors are actually expressing sufficient BRI1 to activate downstream BR signaling? The absence of a rescue could reflect insufficient BRI1 expression in sectors. It will be great if the authors could provide evidence that BRI1-VENUS-expressing sectors show detectable activation of BR signaling (e.g. using a BR signaling reporter?), or at least discuss this limitation.

We apologise for the confusion from our original statement. BRI1-VENUS was dim in all conditions, including after constitutive induction, which rescued leaf size. Therefore, Tau gating was used to image BRI1-VENUS in all cases. We have now made this clear in the methods, by stating: *"Although constitutive induction rescued leaf size, BRI1-GFP fluorescence was dim and often only visible at 20x magnification with tau gating. Therefore, clonal sectors were identified by absence of RFP fluorescence."* (line 459-461).

2. Figure 7 discrepancy: The text (line 253-254) states spongy porosity was reduced in AtML1::BB, but the title of Figure 7 says "reduces palisade, but not spongy mesophyll porosity." This contradiction needs resolving — the data in Fig. 7C shows statistically significant reduction in spongy porosity too, making both the figure title and the result potentially misleading.

To bring the figure title in line with the text, we have now changed the title of figure 7 to *"Repression of epidermal growth reduces mesophyll porosity" (line 685)*

3. Lines 257-258, the authors state that the AtML1::BB data show that "reducing epidermal growth can reduce air space expansion in the mesophyll tissue non-cell autonomously, independently of stomata." This conclusion is stated too confidently. It is true that AtML1::BB increases stomatal density (Fig. S4A), however the paper does not demonstrate that air space reduction in AtML1::BB is independent of stomata. Please soften the statement.

© 2026. Published by The Company of Biologists under the terms of the Creative Commons Attribution License (https://creativecommons.org/licenses/by/4.0/).

We have replaced with with the softer statement “These data suggest that reducing epidermal growth may reduce air space expansion in the mesophyll tissue non-cell autonomously, independently of stomata” (Lines 260-262)

4, Line 433 refers to "CW0008" which appears to be an error for "CW0153" (the inducible BRI1 sector line). The entire Methods section should be checked carefully for similar errors.

Thank you for noticing this. We have now made this correction and checked the entire methods for similar errors.

Third decision letter

MS ID#: dev.205110R2

MS TITLE: Brassinosteroid controls leaf air space patterning non-cell autonomously by promoting epidermal growth

AUTHORS: James M. Fitzsimons, Ana B. Rock, Richard L. De Falbe, Samantha Fox and Chris D. Whitewoods

Dear Dr Whitewoods (Chris)

I am happy to tell you that your manuscript has been accepted for publication in Development, pending our standard publication integrity checks.

© 2026. Published by The Company of Biologists under the terms of the Creative Commons Attribution License (https://creativecommons.org/licenses/by/4.0/).
